# The shaping and functional consequences of the dosage effect landscape in multiple myeloma

**DOI:** 10.1186/1471-2164-14-672

**Published:** 2013-10-02

**Authors:** Mehmet K Samur, Parantu K Shah, Xujun Wang, Stéphane Minvielle, Florence Magrangeas, Hervé Avet-Loiseau, Nikhil C Munshi, Cheng Li

**Affiliations:** 1 Department of Biostatistics and Computational Biology, Dana-Farber Cancer Institute and Harvard School of Public Health, Boston, MA 02215, USA; 2 Department of Biostatistics and Medical Informatics, Akdeniz University, Antalya, Turkey; 3 Department of Bioinformatics, School of Life Sciences and Technology, Tongji University, Shanghai, China; 4 Inserm UMR892, CNRS 6299, Université de Nantes; Centre Hospitalier Universitaire de Nantes, Unité Mixte de Genomique du Cancer, Nantes, France; 5 Department of Medical Oncology, Dana-Farber Cancer Institute and Harvard Medical School, VA Boston Healthcare System, Boston, MA 02215, USA

**Keywords:** Copy number alteration, Dosage effect, Multiple myeloma, Hyperdiploid, Integrative genomics

## Abstract

**Background:**

Multiple myeloma (MM) is a malignant proliferation of plasma B cells. Based on recurrent aneuploidy such as copy number alterations (CNAs), myeloma is divided into two subtypes with different CNA patterns and patient survival outcomes. How aneuploidy events arise, and whether they contribute to cancer cell evolution are actively studied. The large amount of transcriptomic changes resultant of CNAs (dosage effect) pose big challenges for identifying functional consequences of CNAs in myeloma in terms of specific driver genes and pathways. In this study, we hypothesize that gene-wise dosage effect varies as a result from complex regulatory networks that translate the impact of CNAs to gene expression, and studying this variation can provide insights into functional effects of CNAs.

**Results:**

We propose *gene-wise dosage effect score* and *genome-wide karyotype plot* as tools to measure and visualize concordant copy number and expression changes across cancer samples. We find that dosage effect in myeloma is widespread yet variable, and it is correlated with gene expression level and CNA frequencies in different chromosomes. Our analysis suggests that despite the enrichment of differentially expressed genes between hyperdiploid MM and non-hyperdiploid MM in the trisomy chromosomes, the chromosomal proportion of dosage sensitive genes is higher in the non-trisomy chromosomes. Dosage-sensitive genes are enriched by genes with protein translation and localization functions, and dosage resistant genes are enriched by apoptosis genes. These results point to future studies on differential dosage sensitivity and resistance of pro- and anti-proliferation pathways and their variation across patients as therapeutic targets and prognosis markers.

**Conclusions:**

Our findings support the hypothesis that recurrent CNAs in myeloma are selected by their functional consequences. The novel dosage effect score defined in this work will facilitate integration of copy number and expression data for identifying driver genes in cancer genomics studies. The accompanying R code is available at http://www.canevolve.org/dosageEffect/.

## Background

**Figure 1:**
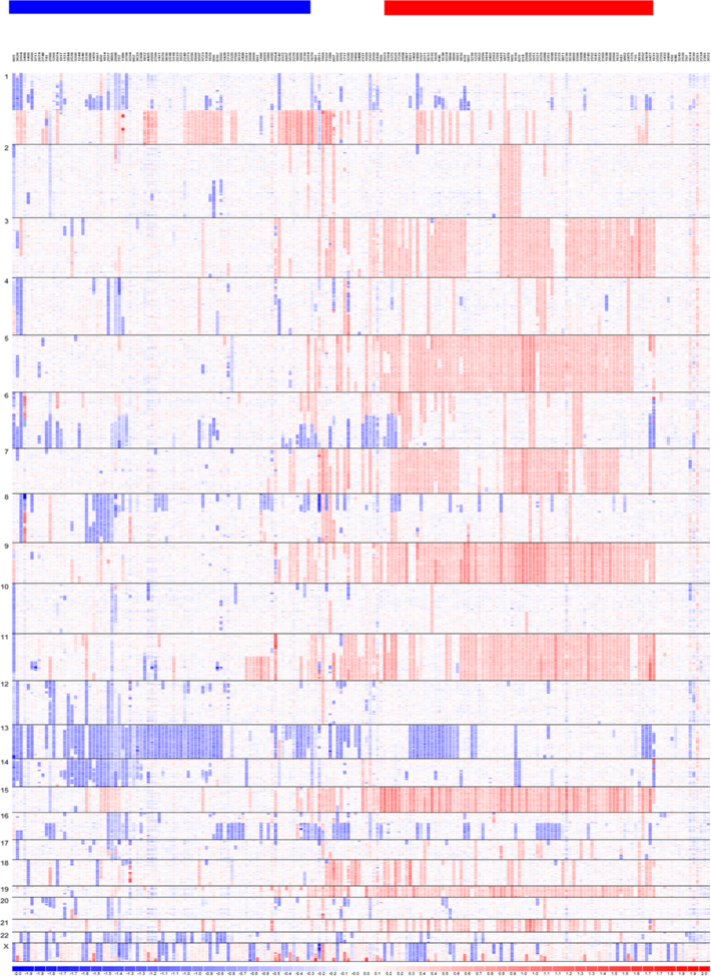
**SNP copy number-based clustering provides an overall view of myeloma genomic heterogeneity and subtypes.** Myeloma samples in the IFM dataset are clustered based on their SNP-array copy numbers from left to right. SNPs are ordered by chromosome positions from top to bottom. Blue color indicates copy number loss, white color indicates copy number close to normal, and red color indicates copy number gains. The horizontal bars on the top indicate samples clusters with copy number features of NHMM (blue) and HMM (red).

Multiple myeloma (MM or myeloma) is a malignant proliferation of plasma B cells that contain prevalent genomic alterations [1,2]. Based on copy number alterations and translocations, myeloma is divided into two main subtypes. The hyperdiploid multiple myeloma (HMM) is characterized by the trisomy (three copies) of eight chromosomes, 3, 5, 7, 9, 11, 15, 19 and 21, and the non-hyperdiploid multiple myeloma (NHMM) is often associated with chromosome 13 hemizygous deletion and translocations between the immunoglobulin heavy chain gene and proto-oncogenes [3]. The copy number alterations (CNAs) in other chromosome regions such as 1q, 6q, 8p, and 16q occur in both subtypes (Figure 1).

Excessively present in myeloma, CNAs are also frequent genomic alteration events in most hematological cancers and many solid cancers [4]. How CNAs arise and whether they contribute to cancer cell evolution is actively debated [5,6]. Some argue that CNAs are a benign side effect of genomic instability during cancer development, which results in many types of mutations and chromosome mis-segregation [7]. Others propose that CNAs have functional consequences on survival and proliferation of cancer cells [8,9], and therefore could be subjected to selection during cancer cell evolution. The eight non-random trisomy chromosomes that characterize HMMs, and better patient survival rates in HMM than in NHMM support the functional selection of CNAs in myeloma [10]. More substantial analysis is needed to identify candidate driver genes or pathways affected by CNAs and design experiments that interfere with these candidates to study their functional effects on cancer cells. However, thousands of genes located in the CNA regions in myeloma have altered mRNA expression levels directly induced by copy number changes, a phenomenon termed copy number dosage effect [11]. In addition, among the genes with dosage effect, transcription factors and epigenetic modifiers could further modulate mRNA expression of their many downstream genes [12]. These large number of directly and indirectly affected genes by CNAs pose big challenges for identifying functional consequences of CNAs in myeloma in terms of specific driver genes and pathways, and for studying the interplay between these driver genes and other genetic mutations during myeloma cell evolution.

Technological development in genomics has promoted multi-scale and integrative studies of cancer [13]. In particular, paired gene expression and copy number profiling for the same cancer samples has helped identifying driver cancer genes in CNA regions, expression signatures that correlate with particular CNAs, and new cancer subtypes [4,14-16]. Copy number dosage effect has been a direct or implied question in such studies, which have shown the dosage effect of genes in various cancer types such as leukemia, lymphoma, liver cancer and breast cancer [17-20]. In myeloma, Dickens and colleagues have identified 170 genes with homozygous deletions and corresponding loss of expression using paired gene expression and copy number alteration profiles [21]. Cell death network is over-represented in these genes, and patients with these deletions have worse overall survival. Agnelli and colleagues have compared myeloma samples with and without chromosome 13 deletions and identified 67 down-regulated genes of which 44 maps to chromosome 13 [22]. These genes are involved in protein biosynthesis, ubiquitination and transcriptional regulation. Many genes and pathways are differentially expressed between the HMM and NHMM subtypes, which have distinct CNA profiles [23,24]. These results support that CNAs have functional consequences through affecting gene expression and may contribute to myeloma cell evolution through cancer-related pathways.

When the extent of CNAs spans multiple chromosomes and hundreds or thousands of genes, such as in myeloma, current dosage effect studies integrating gene and copy number data have limitations. First, they tend to identify pathways with broad functions such as proliferation and apoptosis, whose gene members are enriched in the genes with dosage effect. Although these pathways provide insights into possible contribution of CNAs to cancer, they do not suggest specific therapeutic targets to improving current treatment regimes. Second, regulatory feed-back loops and dosage compensation mechanisms may attenuate the dosage effect of many genes [25], causing them missed by correlation analysis between copy number and expression or differential expression analysis between CNA groups.

Based on these observations, we hypothesize that gene-wise dosage effect varies as a result from complex regulatory networks that translate the impact of CNAs to gene expression, and studying this variation can provide insights into functional effects of CNAs. We propose a gene-wise dosage effect score to measure the prevalence of concordant copy number and expression changes across the samples in a dataset. Applied to two myeloma datasets, a genome-wide karyotype plot of the dosage effect reveals the variation of dosage effect between genes and across chromosomes. We then study the potential causes of these variations in terms of gene’s overall expression level and chromosomes’ CNA frequency. Unexpectedly, four non-trisomy chromosomes (13, 14, 16, and 22) emerge as having the highest proportion of their genes that are dosage sensitive. Dosage sensitive genes and dosage resistant genes are enriched with different functional pathways. In particular, the apoptosis pathway is enriched in dosage resistant genes, raising interesting hypothesis about the mechanism of this phenomenon and its utility as a prognosis marker. Taken together, our findings support that recurrent CNAs in myeloma are selected by their functional consequences. The proposed dosage effect analysis method will facilitate many genomics studies integrating gene copy number and expression data.

## Methods

### Microarray datasets

We used two datasets providing paired genome-wide profiles of copy number alterations and gene expression for newly diagnosed myeloma patients. The first is the IFM (Intergroupe Francophone du Myelome) dataset with 170 patients profiled in our laboratory by high-density Affymetrix 500 K SNP arrays and Exon ST 1.0 expression arrays (GEO identifiers GSE12896 and GSE39754). Patient characteristics and clinical information have been reported previously [2]. The second is the Dickens dataset with 71 patients profiled by Affymetrix 500 K SNP arrays and Human Genome U133 Plus 2.0 expression arrays (GSE15695) [21].

### Primary analysis of gene expression and copy number data

The gene expression and copy number profiling data were preprocessed with the dChip software [26,27] to obtain expression and copy number estimates. The gene expression profiles were quantile-normalized and model-based signals were computed [26,27]. Gene probe set intensity values were averaged when more than one probe sets per gene were available. To assess the impact of alternative methods for handling Affymetrix array probe sets on Dosage effect scores, we also obtained alternative expression estimates for the IFM dataset from a CDF (HuEx-1_0-st-v2,coreR3,A20071112,EP.CDF) provided by Purdom *et. al.*[28]. This CDF, available form aroma.affymetrix website, groups Affymetrix “core” probesets into 18,708 transcript clusters. In this setting, each exon and probe is uniquely mapped to only one transcript cluster, allowing for a unique gene expression value for each individual gene after pre-processing and eliminating the need to use avarage value for each gene.

A gene’s copy number was obtained by averaging the copy numbers of the SNPs within 5 kb of the gene’s transcription region. Further analysis was done with the R programming environment. We created gene expression and copy number data matrices based on unique gene symbols, and then identified the gene symbols common between the two data matrices for dosage effect analysis: 12836 genes for the IFM dataset and 12359 genes for the Dickens dataset.

### Hyperdiploidy status

Since there were no published hyperdiploidy data determined by FISH for both datasets, the myeloma hyperdiploidy status was defined using the copy number information from SNP arrays. To determine the threshold for separating samples in to two subtypes, we plotted the distribution of the median copy number of the trisomy chromosomes. We observed a bimodal distribution with two peaks at 2 and 2.6. The HMM samples have a peak near 2.6 (and not 3) due to the normalization effect across arrays. We chose the midway value (2.3) between the two peaks as the cutoff to call HMM and NHMM samples from the copy number data. 77 of 170 IFM samples, and 42 of 71 Dickens samples were called as HMM, and the rest samples were called as NHMM.

### Defining dosage effect score (DES)

We defined a gene-wise dosage effect score (DES) to identify genes whose copy number changes modulate their own expression. The DES is the ratio between “dosage effect sample number” and “CNA sample number”. The “CNA sample number” of a gene, is the number of samples in a dataset that have CNA at the gene (copy number < 1.7 or > 2.3, considering signal noise and normal cell contamination). The “dosage effect sample number” of the gene is defined using two steps. First, using the samples with no copy number changes at the gene (copy number within 1.7 and 2.3), the mean (normal_mean) and standard deviation (normal_sd) of the gene expression values is calculated. Next, we count the total number of samples that have CNA at this gene and their expression levels at the gene change more than random in the same direction as their CNA (i.e. the samples whose gene expression value are higher than normal_mean + normal_sd for amplification samples or lower than normal_mean – normal_sd for deletion samples). The number of these samples is the “dosage effect sample number” at the gene.

Being a ratio, DES ranges between 0 and 1. Larger DES indicates stronger dosage effect at the gene in the sense that a larger proportion of CNA samples have concordant expression changes. To ensure reliability, DES is computed only for genes whose CNA sample number is greater than 10% of all samples. As an example, the gene EIF3K has CNA in 55.88% of samples in the IFM data set. Utilizing mean +/− 1 SD criteria, the percent of samples with both copy number alteration and concordant expression changes are 25.29%. EIF3K has the DES of 0.45 (.2559/.5588) in the IFM dataset.

### Differential and functional enrichment analysis

Differential expression was identified with the *LIMMA* Bioconductor package, and Benjamini-Hochberg multiple hypothesis correction was carried out using the R package *multtest*. Differential gene expression was called at the adjusted p-value < 0.05. We used the DAVID web server for gene function enrichment analysis using gene ontology (GO) categories (GOTERM_BP_FAT, GOTERM_CC_FAT and GOTERM_MF_FAT) [29].

## Results

### Differentially expressed genes between HMM and NHMM are enriched in the trisomy chromosomes

**Figure 2:**
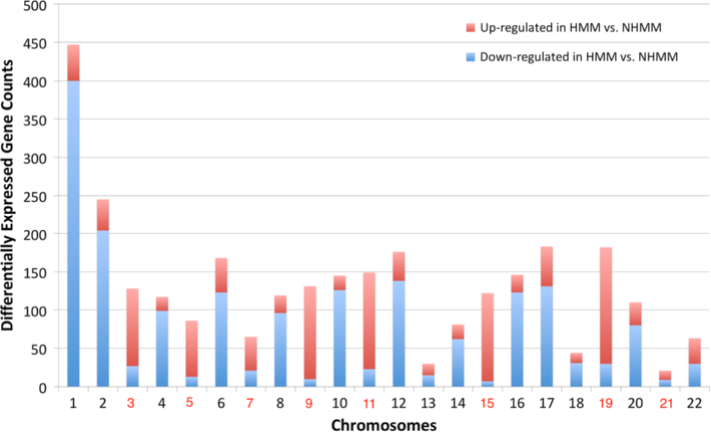
**The number of differentially expressed genes between HMM and NHMM by chromosome.** Red and blue bars represent up-regulated genes and down-regulated genes in HMM samples compared to NHMM samples, respectively. No normal samples are available or used for this dataset. On the X-axis label, trisomy chromosomes in HMM are colored in red.

A primary difference between the HMM and NHMM subtypes is the presence of three copies (trisomy) of eight specific chromosomes in HMM and normal copy numbers for them in NHMM (Figure 1). To check whether the trisomy chromosomes influence the expression of the genes located within them, we compared the HMM and NHMM samples of the IFM dataset for differentially expressed genes. The trisomy chromosomes contained more HMM up-regulated genes than HMM down-regulated genes (Figure 2). Specifically, 744 of the 4702 (15.8%) trisomy chromosome genes are up-regulated in HMM, while only 416 of the 8134 (5.1%) non-trisomy chromosome genes are up-regulated in HMM (Fisher’s exact test p-value < 2.2e-16).

In contrast, genes down-regulated in HMM are significantly depleted from the trisomy chromosomes (140 of 1798 down-regulated genes locate in the trisomy chromosomes, p-value < 2.2e-16). Such enrichment is not due to that expressed genes in myeloma preferentially locate in the trisomy chromosomes. The reason is as follows: when the expressed genes in myeloma is defined as the top 50% genes ranked by median expression level across the IFM samples, 51.4% (2418 genes) of the trisomy chromosome genes are expressed and 49.2% (3999 genes) of the non-trisomy chromosomes are expressed (p-value = 0.014).

Chromosome 1q amplification is observed in ~40% of myeloma patients (e.g. Figure 1) and many genes on 1q21 have been reported as over-expressed when comparing myeloma against normal. In the IFM data, we observe much more down-regulated genes than up-regulated genes from chromosome 1 when comparing HMM vs. NHMM samples (Figure 2). This can be due to more frequent amplification of chromosome 1q in NHMM than in HMM (Figure 1). These results suggest that chromosome copy number changes in myeloma could have direct effect on the expression level of a substantial number of genes.

### Widespread but variable dosage effect in myeloma

**Figure 3:**
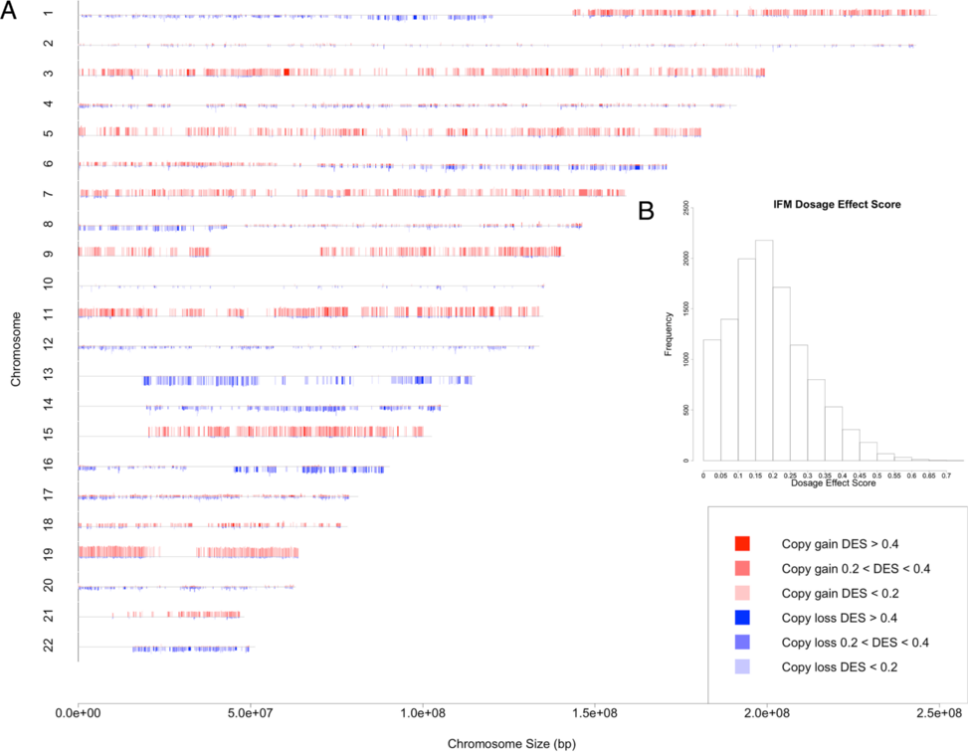
**The CNA frequency and dosage effect karyotype plot for the IFM dataset. (A)** The chromosomes are numbered from 1 to 22 and marked by gene locations. For each gene, the height of red and blue bars represents the percent of samples with copy number gain and copy number loss, respectively. The height of gray bars at the beginning of chromosomes indicates 100%. The colors represent the dosage effect score (DES) of genes, which measures concordant copy number and expression changes. **(B)** The distribution of gene dosage effect scores.

**Figure 4:**
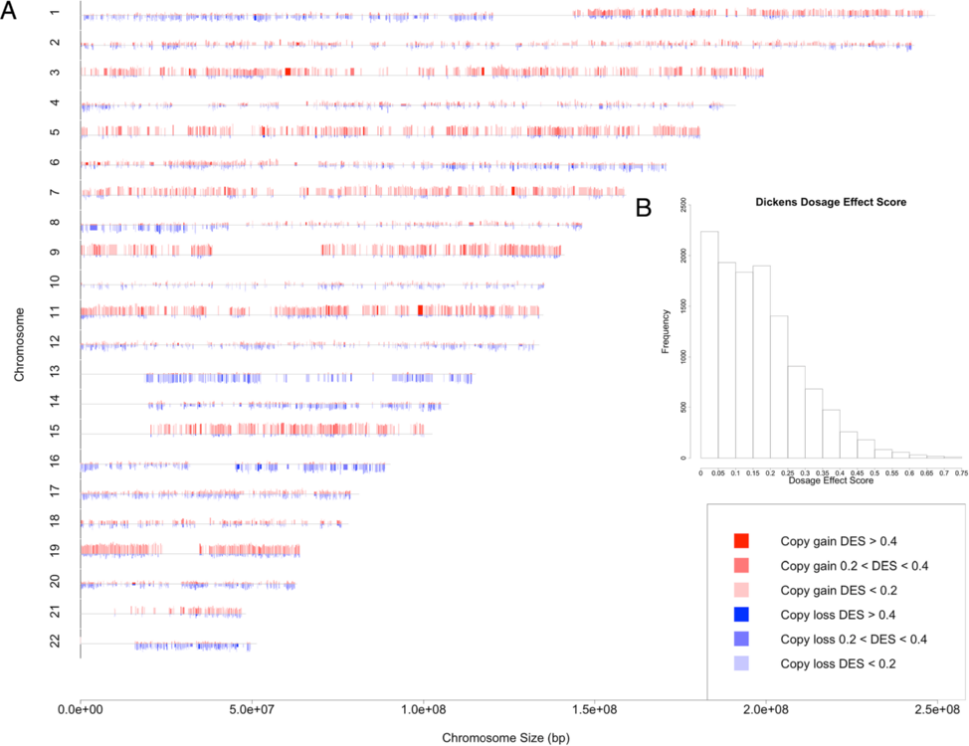
**The CNA frequency and dosage effect karyotype plot for the Dickens dataset. (A)** The chromosomes are numbered from 1 to 22 and marked by gene locations. For each gene, the height of red and blue bars represents the percent of samples with copy number gain and copy number loss, respectively. The height of gray bars at the beginning of chromosomes indicates 100%. The colors represent the dosage effect score (DES) of genes, which measures concordant copy number and expression changes. **(B)** The distribution of gene dosage effect scores.

To understand the global and spatial patterns of how copy number alterations (CNAs) affect gene expression in myeloma, we integrated paired gene expression and copy number profiling data from two myeloma datasets. We defined a dosage effect score (DES) for each gene as the proportion of CNA samples that have concordant copy number and gene expression changes (see Methods), and then visualized the gene-wise CNA frequencies and DES in a genome-wide karyotype plot (Figures 3A and 4A). The plots show that the dosage effect in myeloma patients is widespread and there is an overall similar dosage effect patterns between the IFM and Dickens datasets. The impact of dosage on gene expression can be observed at most chromosomes, such as the trisomy chromosomes 9, 11 and 15, and chromosomes 13, 14 and 16, which contain hemizygously deleted regions.

DES ranges between 0 and 1, and summarizes concordant copy number and gene expression changes. A value close to 0 and 1 indicates weak and strong dosage effect, respectively. The distribution of DES in the IFM data shows a large variation of DES across genes: 58% genes have low dosage effect (DES < 0.2, Figure 3B), 37% have medium dosage effect (DES between 0.2 and 0.4), and only 5.2% (611 genes) have high dosage effect (DES > 0.4). For the Dickens data the distribution is similar: 64%, 30%, and 5.5% (655) genes have low, medium and high dosage effect, respectively (Figure 4B). The high dosage effect genes from the two datasets have 123 genes in common (Fisher’s exact p-value < 2.2e-16). The top 100 dosage-sensitive as well as resistant-genes for IFM and Dickens datasets are reported in Additional files 1 and 2. Deletion in chromosome 13 is a prognostic factor in MM [3]. Therefore genes showing strong dosage effect on this chromosome may be important for MM. A list of dosage sensitive genes on this chromosome is available in Additional file 3. ELOVL7, PTDSS1, POLR1D, TBC1D22A, CCNC and TCF25 are some of the interesting genes with cancer related functions identified from the DES analysis. Additional file 4 summarizes cancer related functions based on PubMed for top 50 dosage sensitive genes in both IFM and Dickens datasets.

### Higher gene expression level is associated with stronger dosage effect

**Figure 5:**
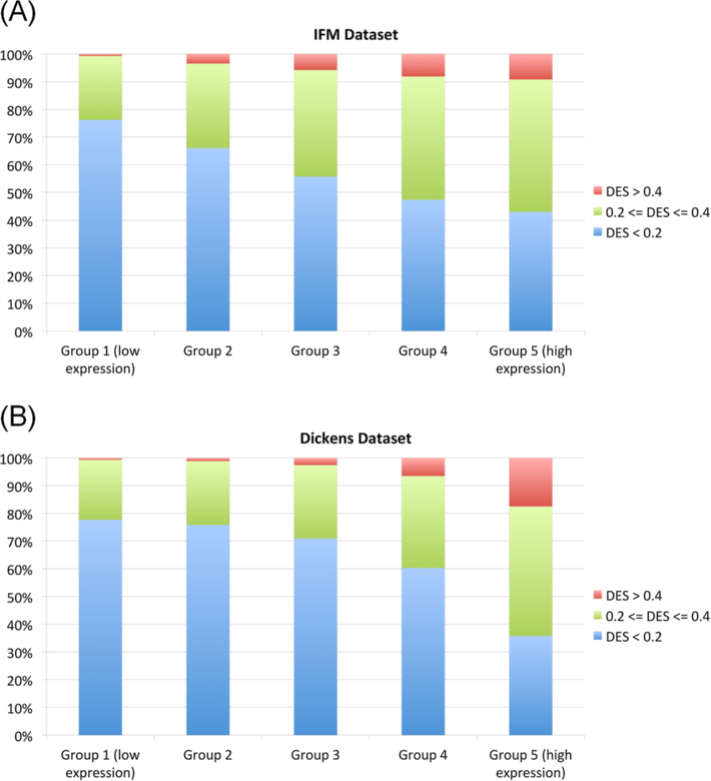
**The correlation between dosage effect and gene expression level. (A)** The IFM dataset, and **(B)** the Dickens dataset. Group 1 includes the 20% genes that have lowest overall expression across samples, and Group 5 includes the 20% genes that have highest overall expression.

To explain the variation of dosage effect for the genes with similar CNA frequencies, such as those in the same trisomy chromosome (Figure 3A), we correlated gene expression levels with the DES. The motivation is that some genes are expressed at higher level than others, and genes that are not expressed in myeloma will not exhibit any dosage effect. We first ordered all the genes by their median expression levels across samples and divided the genes into five equal-size groups (Figure 5, X-axis), and then compared the proportion of genes having low, medium and high dosage effect across the groups (Figure 5, Y-axis). We found that for both datasets, higher expressed gene groups contain significant larger proportion of genes with high dosage effect (Cochran-Armitage p-value < 2.2e-16 for both datasets).

### Higher chromosomal CNA frequency is correlated with higher proportion of dosage sensitive genes

**Figure 6:**
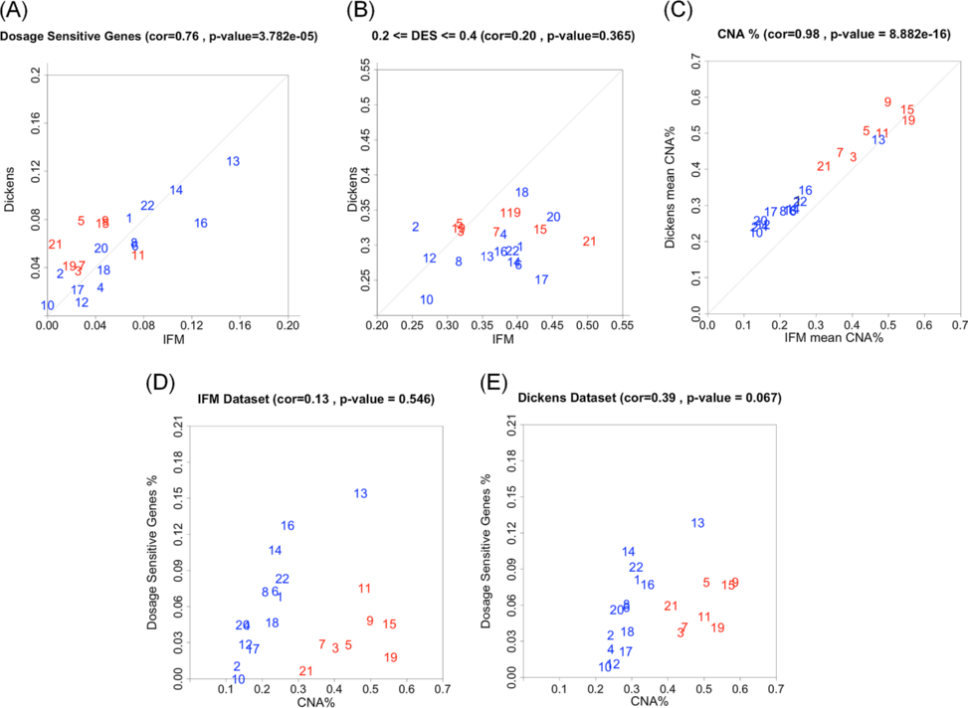
**The correlation between dosage sensitive genes and CNA frequency across chromosomes. (A)** The proportion of genes in each chromosome that are dosage sensitive (DES > 0.4). X-axis: the IFM dataset, Y-axis: the Dickens dataset. **(B)** The proportion of genes in each chromosome that have medium dosage effect (0.2 < = DES < = 0.4). X-axis: the IFM dataset, Y-axis: the Dickens dataset. **(C)** Copy number alteration frequency in each chromosome compared between the two datasets. Each point represents the CNA (copy number <1.7 or > 2.3) frequency of a gene across samples, averaged over all the genes in a chromosome. **(D, E)** The proportion of dosage sensitive genes (Y-axis) is plotted against the average CNA frequency (X-axis) for different chromosomes, for the IFM data **(D)** and the Dickens data **(E)**.

The proportion of dosage sensitive genes (DES > 0.4) in each chromosome also varies across chromosomes, ranging from about 2% for chromosome 17 to >12% for chromosome 13 (Figure 6A). These proportions strongly correlate between the two datasets. Interestingly, the chromosomal proportion of dosage sensitive genes is highest for four non-trisomy chromosomes (chromosomes 13, 14, 16 and 22) and lower for the trisomy chromosomes (marked red in Figure 6A). In contrast, the proportion of genes with medium dosage effect in each chromosome is not correlated between the two datasets (Figure 6B).

Because the pattern of recurrent chromosomal gains and losses in myeloma are conserved across different datasets (Figures 1 and 6C), we asked whether the variation in the proportion of dosage sensitive genes across chromosomes is correlated with the myeloma CNA patterns. We compared the proportion of dosage sensitive genes with the proportion of genes with CNA across chromosomes. For non-trisomy chromosomes (marked blue in Figure 6D, 6E), we observed that larger CNA proportions associate with higher proportion of dosage sensitive genes.

### Both dosage sensitive genes and differentially expressed genes between HMM and NHMM are enriched by protein translation and localization processes

To explore the functional consequences of dosage effect, we performed functional enrichment analysis for dosage sensitive genes (DES > 0.4, genes listed in Additional file 1 and 2 for the IFM and Dickens dataset respectively) using the DAVID gene ontology Web server. Translation, protein localization, and proteolysis processes are enriched in dosage sensitive genes from the IFM dataset (Additional file 5: Table S1). Translation, protein localization and mitochondrial processes are enriched in dosage sensitive genes from the Dickens dataset (Additional file 5: Table S2).

Since the distinct CNA patterns in the HMM and NHMM subtypes can induce subtype specific expression changes through dosage effect, we asked how dosage effect analysis compares to differential expression analysis. We compared 2958 differentially expressed genes between HMM and NHMM (Figure 2) and 611 dosage sensitive genes (DES > 0.4) from the IFM dataset. Their overlap of 306 genes is highly significant (Fisher’s exact test p-value < 2.2e-16). Gene ontology groups enriched in HMM up-regulated genes include translation and RNA-binding, and those enriched in NHMM up-regulated genes include protein localization, proteolysis, cell cycle, RNA processing and histone modification (Additional file 5: Table S3). These results suggest that differentially expressed genes between the two myeloma subtypes are mainly driven by dosage sensitive genes located in distinct CNA regions of the two subtypes.

### Dosage resistant genes are enriched by the apoptosis process

Compared to dosage sensitive genes, dosage resistant genes (Additional file 1 and Additional file 2) may be subjected to more stringent dosage compensatory networks such as feedback loops. Dosage resistant genes were defined as the top 20% of highly expressed genes (Group 5 from Figure 5) that have DES < 0.2 (genes listed in Additional files 1 and 2). Gene ontology enrichment analysis of dosage resistant genes identified processes such as translation and organelle lumen, which are commonly enriched in both dosage sensitive and resistant genes. However, *apoptosis* emerged as a distinct process only enriched in dosage resistant genes in both datasets (Additional file 5: Table S4, Table S5).

### Alternative pre-processing of Affymetrix Exon arrays does not impact dosage effect score substantially

It is valuable to assess impact of alternative methods for handling Affymetrix Exon array probe sets on DES. Therefore, we obtained alternative expression estimates for the IFM dataset from a CDF provided by Purdom *et al*. [28]. For the IFM dataset, the Pearson correlation coefficient for the mean of gene expression estimates across the samples of *dChip* and *aroma.affymetrix* normalized data is 0.86 (12192 genes; p < 2.2e-16) and the correlation for DES scores is 0.81 (10960 genes; p < 2.2e-16). Moreover, the results from functional enrichment analysis of DES scores from the IFM dataset derived using aroma.affymetrix expression estimates overlap significantly with those obtained from dChip expression estimates (Additional file 5: Figure S1A and S1B, Additional file 5: Table S6 and S7). In addition, we can observe similar DES patterns for chromosomal locations (Figure 3 and Additional file 5: Figure S2). Please see Additional file 5 for detailed enrichment results. Thus, conclusions from our analysis are robust against different pre-processing methods.

## Discussion

Cancer genomes such as those of multiple myeloma harbor many types of genomic aberrations. Among them, CNAs of entire chromosomes or focal chromosomal regions have been extensively detected from many cancer types in the past decade through microarrays and massively parallel sequencing technologies. Paired copy number and gene expression profiling of the same cancer samples has enabled integrative analysis identifying driver oncogenes, improving classification of cancer subtypes, and provided better understanding of molecular pathways dysregulated in cancer [4,15,19,30]. In myeloma genomes, prevalent and recurrent patterns of 8 trisomy chromosomes and deletions of specific chromosomes (1p, 6q, 8p, 13, 16q; see Figure 1) provide a model system to study the effect of CNAs on gene expression. Although previous studies hinted at the copy number dosage effect in various cancer types including myeloma [11,18], in this study we have for the first time defined dosage effect score, highlighted its variation across genes and chromosomes, and studied the potential consequence of these variations in terms of dosage sensitive genes and dosage-resistant genes. Other genomic changes such as copy-neutral loss of heterozygosity can be inferred from SNP array data, and they could affect gene expression levels. Study of these alterations can be followed up in the future.

Defining a gene-wise dosage effect score (DES) allows us to compare and visualize the impact of CNAs on gene expression at the level of individual genes, across the genome and between datasets. The DES score was calculated only for those genes that have copy number alterations in at least 10% of patients and vary their expression beyond the mean expression +/− one SD, as a safeguard in the process. Because cis-regulatory factors may reduce the gene expression output even if the copy number has increased, using higher SD values could result in significantly higher amount of false negatives. In practice, users of our method can use any suitable threshold of choice. The visualization of distribution of DES along chromosomes suggests that dosage effect in myeloma is widespread and variable across the genome (Figures 3 and 4). Most genes show low or moderate dosage effect, and only about 5% of genes are highly dosage sensitive. We analyze two contributing causes for such variation: highly expressed genes are more likely to have higher dosage effect (Figure 5), and higher frequency of CNAs in a chromosome correlates with higher proportion of dosage sensitive genes (Figure 6D and 6E). Interestingly, although three non-trisomy chromosomes (14, 16, 22) have lower frequency of CNAs than the 8 trisomy chromosomes, the proportion of dosage sensitive genes in these non-trisomy chromosomes are higher than in the trisomy chromosomes (Figure 6D and 6E). This could be due to that 3-copy gains in trisomy chromosomes lead to 1.5 fold expression changes, while 1-copy losses in non-trisomy chromosomes lead to 2 fold expression changes. The larger relative impact on gene expression of copy number losses may lead to higher proportion of dosage sensitive genes in non-trisomy chromosomes.

Dosage sensitive genes are enriched by genes with function in protein translation and localization. Recently, dosage effects of protein translation and transport genes are reported for plasma cell leukemia, a cancer closely related to myeloma [31]. Overdrive of translation machines is a feature of cancer cell proliferation [32-34]. Dosage sensitivity of the genes in translation processes indicates their expression levels are not subject to tightly controlled dosage compensation and are susceptible to the influence of copy number alteration. These results support that CNA patterns in myeloma, such as the eight characteristic trisomy chromosomes in about half of all myeloma patients, are not a by-product of myeloma genomic evolution but have functional and consequential effects that contribute to the pathogenesis of myeloma.

We find that gene ontology groups enriched in dosage sensitive genes are similar to those enriched in the differential expressed genes between HMM and NHMM subtypes (Additional file 5: Tables S1, S2 and S3), and these two gene sets overlap significantly. Previous gene expression studies comparing HMM and NHMM have found similar enriched pathways. Chng *et. al*. reported over-expression of ribosomal protein and protein biosynthesis genes in HMM relative to NHMM [24]. Agnelli *et. al*. reported that up-regulated genes in HMM compared to NHMM are mainly involved in protein biosynthesis, encoding for ribosomal proteins and mitochondrial ribonucleoproteins [23], and these genes mainly mapped to the hyperdiploid chromosomes. Therefore, distinct CNA patterns in HMM and NHMM are likely to be the cause for most differentially expressed genes between the two subtypes through dosage effect.

We also identify specific dosage sensitive genes that may play critical roles in myeloma development. For example (Additional files 1, 2 and 3), Tamura *et. al*. [35] showed that EVOLV7 could be involved in prostate cancer growth and survival. Camps *et. al*. [36] identified that loss of POLR1D function affects cell viability in colorectal cancers. Cell cycle role of CCNC and its effect on several cancer types were reported by several authors [37,38]. Steen *et. al*. previously reported the role of NULP1 (TCG25) in cell death control and tumor growth [39]. Additional file 4 includes literature search for the top 50 dosage sensitive genes for both IFM and Dickens dataset.

A less studied phenomenon in integrative genomics is dosage resistance or compensation. Essential genes whose expression level must be tightly regulated may achieve dosage resistance through feedback loops that compensate for copy number changes [40]. A recent study reports that amplified genes may be over-expressed, unchanged or down-regulated in cervical cancer [41]. Signaling and receptor activity functions are enriched in genes whose expression negatively correlates with copy number in glioblastoma [42]. In our analysis, we found apoptosis as an enriched category in dosage resistant genes but not in dosage sensitive genes. The gene ontology category “apoptosis” encompasses a large number of genes with both “signaling” and other functions. This ontology as any categorization is subjective and incomplete with gaps in knowledge. The apoptosis pathway is often mutated or inhibited in cancer cells for its tumor suppressing function [43]. Dickens *et al*. have identified 170 “cell death” signature genes whose homozygous deletions and corresponding loss of expression in myeloma are adverse to overall survival [21]. These results point to future studies on differential dosage sensitivity and resistance of pro- and anti-proliferation pathways and their variation across patients as therapeutic targets and prognosis markers.

There are several limitations of this study. First, we haven’t considered indirect effects of CNAs. CNAs could directly alter the expression level of transcription factors and signaling transduction genes, which in turn affect their downstream genes’ expression. Partly alleviating this limitation, our identification of dosage-resistant genes may be reflection of indirect effects. Second, parameters and thresholds used in the analysis are chosen based on empirical distributions and consideration for reducing noise, such as computing DES only for genes with CNA frequency >10% and regarding genes as dosage-sensitive at DES > 0.4, but choosing other parameters may lead to different results. We have used DSE > 0.3 to select dosage sensitive genes and obtained similar enriched pathways in dosage sensitive genes. The agreement between the two myeloma datasets with different patient cohorts also supports the robustness of the analysis method and parameters used. These limitations point to directions for further improvement of dosage effect analysis.

## Conclusions

In summary, dosage effect score is a gene-wise measure that integrates copy number alteration with gene expression in a quantitative manner. It could be an important tool to understanding the functional impact of CNAs in tumor development. Our results indicate that CNAs in myeloma impact the gene expression level of a substantial number of genes. Interestingly, our analysis suggests that even though differentially expressed genes between HMM and NHMM are enriched in the trisomy chromosomes, chromosomal proportion of dosage sensitive genes is highest for non-trisomy chromosomes. Also, higher expression levels associated with stronger dosage effect. The dosage sensitive genes are enriched by protein translation and localization processes, and dosage resistant genes are enriched by genes with function in apoptosis. These results support that CNAs exert functional effect in cancer transcriptome in part through direct dosage effect of CNA-affected genes, and therefore recurrent CNAs can be selected during cancer clonal evolution due to their dosage effect on specific pathways. The gene lists identified in our analysis will help identify functional targets in myeloma. The R code is available at http://www.canevolve.org/dosageEffect/ for applying this method to other studies profiling paired gene expression and copy number cancer samples.

### Availability of supporting data

All supporting data are included as Additional files 1, 2, 3, 4 and 5. The data sets supporting the results of this article are available in the Gene Expression Omnibus (GEO) repository, GSE12896, GSE39754 and GSE15695 at http://www.ncbi.nlm.nih.gov/geo/.

## Competing interests

The authors declare that they have no competing interests.

## Authors’ contributions

MKS, PKS, NCM and CL designed the study. MKS implemented the methodology. PKS and MKS analyzed the data. SM, FM and HA-L generated the IFM dataset. XW contributed to data analysis. MKS, PKS and CL wrote the manuscript. All authors reviewed and approved the manuscript.

## Supplementary Material

Additional file 1 List of top 100 dosage sensitive and resistant genes for the IFM dataset and dosage effect scores of apoptosis pathway member genes. Click here for file

Additional file 2 List of top 100 dosage sensitive and resistant genes for the Dickens dataset and dosage effect scores of apoptosis pathway member genes. Click here for file

Additional file 3 Genes showing strong dosage effect on chromosome 13 for the IFM and Dickens datasets. Click here for file

Additional file 4 Cancer-related functions of top 50 dosage sensitive genes in the IFM and Dickens datasets. Click here for file

Additional file 5 Supplementary tables and figures. Click here for file

## References

[B1] Anderson KC Carrasco RD Pathogenesis of myeloma Annu Rev Pathol 2011 14 249 274 10.1146/annurev-pathol-011110-130249 21261519

[B2] Avet-Loiseau H Li C Magrangeas F Gouraud W Charbonnel C Harousseau JL Attal M Marit G Mathiot C Facon T Prognostic significance of copy-number alterations in multiple myeloma J Clin Oncol 2009 14 4585 4590 10.1200/JCO.2008.20.6136 19687334 PMC2754906

[B3] Munshi NC Avet-Loiseau H Genomics in multiple myeloma Clin Cancer Res 2011 14 1234 1242 10.1158/1078-0432.CCR-10-1843 21411439 PMC3783001

[B4] Huang N Shah PK Li C Lessons from a decade of integrating cancer copy number alterations with gene expression profiles Brief Bioinform 2011 14 305 316 21949216 10.1093/bib/bbr056 PMC3357489

[B5] Gordon DJ Resio B Pellman D Causes and consequences of aneuploidy in cancer Nat Rev Genet 2012 14 189 203 22269907 10.1038/nrg3123

[B6] Weaver BA Cleveland DW Does aneuploidy cause cancer? Curr Opin Cell Biol 2006 14 658 667 10.1016/j.ceb.2006.10.002 17046232

[B7] Hede K Which came first? Studies clarify role of aneuploidy in cancer J Natl Cancer Inst 2005 14 87 89 10.1093/jnci/97.2.87 15657335

[B8] Holland AJ Cleveland DW Boveri revisited: chromosomal instability, aneuploidy and tumorigenesis Nat Rev Mol Cell Biol 2009 14 478 487 19546858 10.1038/nrm2718 PMC3154738

[B9] Weaver BA Cleveland DW Aneuploidy: instigator and inhibitor of tumorigenesis Cancer Res 2007 14 10103 10105 10.1158/0008-5472.CAN-07-2266 17974949 PMC3132555

[B10] Avet-Loiseau H Attal M Moreau P Charbonnel C Garban F Hulin C Leyvraz S Michallet M Yakoub-Agha I Garderet L Genetic abnormalities and survival in multiple myeloma: the experience of the Intergroupe Francophone du Myelome Blood 2007 14 3489 3495 10.1182/blood-2006-08-040410 17209057

[B11] Agnelli L Mosca L Fabris S Lionetti M Andronache A Kwee I Todoerti K Verdelli D Battaglia C Bertoni F A SNP microarray and FISH-based procedure to detect allelic imbalances in multiple myeloma: an integrated genomics approach reveals a wide gene dosage effect Genes Chromosomes Cancer 2009 14 603 614 10.1002/gcc.20668 19396863

[B12] Rudin CM Durinck S Stawiski EW Poirier JT Modrusan Z Shames DS Bergbower EA Guan Y Shin J Guillory J Comprehensive genomic analysis identifies SOX2 as a frequently amplified gene in small-cell lung cancer Nat Genet 2012 14 1111 1116 10.1038/ng.2405 22941189 PMC3557461

[B13] Cancer Genome Atlas Research N Comprehensive genomic characterization defines human glioblastoma genes and core pathways Nature 2008 14 1061 1068 10.1038/nature07385 18772890 PMC2671642

[B14] Tomlins SA Mehra R Rhodes DR Cao X Wang L Dhanasekaran SM Kalyana-Sundaram S Wei JT Rubin MA Pienta KJ Integrative molecular concept modeling of prostate cancer progression Nat Genet 2007 14 41 51 10.1038/ng1935 17173048

[B15] Garraway LA Widlund HR Rubin MA Getz G Berger AJ Ramaswamy S Beroukhim R Milner DA Granter SR Du J Integrative genomic analyses identify MITF as a lineage survival oncogene amplified in malignant melanoma Nature 2005 14 117 122 10.1038/nature03664 16001072

[B16] Akavia UD Litvin O Kim J Sanchez-Garcia F Kotliar D Causton HC Pochanard P Mozes E Garraway LA Pe’er D An integrated approach to uncover drivers of cancer Cell 2010 14 1005 1017 10.1016/j.cell.2010.11.013 21129771 PMC3013278

[B17] Hungermann D Schmidt H Natrajan R Tidow N Poos K Reis-Filho JS Brandt B Buerger H Korsching E Influence of whole arm loss of chromosome 16q on gene expression patterns in oestrogen receptor-positive, invasive breast cancer J Pathol 2011 14 517 528 10.1002/path.2938 21706489

[B18] Sellmann L Scholtysik R Kreuz M Cyrull S Tiacci E Stanelle J Carpinteiro A Nuckel H Boes T Gesk S Gene dosage effects in chronic lymphocytic leukemia Cancer Genet Cytogenet 2010 14 149 160 10.1016/j.cancergencyto.2010.09.002 21156227

[B19] Monti S Chapuy B Takeyama K Rodig SJ Hao Y Yeda KT Inguilizian H Mermel C Currie T Dogan A Integrative analysis reveals an outcome-associated and targetable pattern of p53 and cell cycle deregulation in diffuse large B cell lymphoma Cancer Cell 2012 14 359 372 10.1016/j.ccr.2012.07.014 22975378 PMC3778921

[B20] Roessler S Long EL Budhu A Chen Y Zhao X Ji J Walker R Jia HL Ye QH Qin LX Integrative genomic identification of genes on 8p associated with hepatocellular carcinoma progression and patient survival Gastroenterology 2012 14 957 966 e912 10.1053/j.gastro.2011.12.039 22202459 PMC3321110

[B21] Dickens NJ Walker BA Leone PE Johnson DC Brito JL Zeisig A Jenner MW Boyd KD Gonzalez D Gregory WM Homozygous deletion mapping in myeloma samples identifies genes and an expression signature relevant to pathogenesis and outcome Clin Cancer Res 2010 14 1856 1864 10.1158/1078-0432.CCR-09-2831 20215539 PMC2841345

[B22] Agnelli L Bicciato S Fabris S Baldini L Morabito F Intini D Verdelli D Callegaro A Bertoni F Lambertenghi-Deliliers G Integrative genomic analysis reveals distinct transcriptional and genetic features associated with chromosome 13 deletion in multiple myeloma Haematologica 2007 14 56 65 10.3324/haematol.10414 17229636

[B23] Agnelli L Fabris S Bicciato S Basso D Baldini L Morabito F Verdelli D Todoerti K Lambertenghi-Deliliers G Lombardi L Neri A Upregulation of translational machinery and distinct genetic subgroups characterise hyperdiploidy in multiple myeloma Br J Haematol 2007 14 565 573 10.1111/j.1365-2141.2006.06467.x 17367409

[B24] Chng WJ Kumar S Vanwier S Ahmann G Price-Troska T Henderson K Chung TH Kim S Mulligan G Bryant B Molecular dissection of hyperdiploid multiple myeloma by gene expression profiling Cancer Res 2007 14 2982 2989 10.1158/0008-5472.CAN-06-4046 17409404

[B25] Birchler JA Reflections on studies of gene expression in aneuploids Biochem J 2010 14 119 123 10.1042/BJ20091617 20141513

[B26] Li C Wong WH Model-based analysis of oligonucleotide arrays: expression index computation and outlier detection Proc Natl Acad Sci USA 2001 14 31 36 10.1073/pnas.98.1.31 11134512 PMC14539

[B27] Lin M Wei LJ Sellers WR Lieberfarb M Wong WH Li C dChipSNP: significance curve and clustering of SNP-array-based loss-of-heterozygosity data Bioinformatics 2004 14 1233 1240 10.1093/bioinformatics/bth069 14871870

[B28] Purdom E Simpson KM Robinson MD Conboy JG Lapuk AV Speed TP FIRMA: a method for detection of alternative splicing from exon array data Bioinformatics 2008 14 1707 1714 10.1093/bioinformatics/btn284 18573797 PMC2638867

[B29] da Huang W Sherman BT Lempicki RA Bioinformatics enrichment tools: paths toward the comprehensive functional analysis of large gene lists Nucleic Acids Res 2009 14 1 13 10.1093/nar/gkn923 19033363 PMC2615629

[B30] Kuijjer ML Rydbeck H Kresse SH Buddingh EP Lid AB Roelofs H Burger H Myklebost O Hogendoorn PC Meza-Zepeda LA Cleton-Jansen AM Identification of osteosarcoma driver genes by integrative analysis of copy number and gene expression data Genes Chromosomes Cancer 2012 14 696 706 10.1002/gcc.21956 22454324

[B31] Mosca L Musto P Todoerti K Barbieri M Agnelli L Fabris S Tuana G Lionetti M Bonaparte E Sirchia SM Genome-wide analysis of primary plasma cell leukemia identifies recurrent imbalances associated with changes in transcriptional profiles Am J Hematol 2013 14 16 23 10.1002/ajh.23339 23044976

[B32] Zismanov V Drucker L Attar-Schneider O Matalon ST Pasmanik-Chor M Lishner M Tetraspanins stimulate protein synthesis in myeloma cell lines J Cell Biochem 2012 14 2500 2510 10.1002/jcb.24126 22415769

[B33] Yamasaki S Anderson P Reprogramming mRNA translation during stress Curr Opin Cell Biol 2008 14 222 226 10.1016/j.ceb.2008.01.013 18356035 PMC2841789

[B34] Silvera D Formenti SC Schneider RJ Translational control in cancer Nat Rev Cancer 2010 14 254 266 10.1038/nrc2824 20332778

[B35] Tamura K Makino A Hullin-Matsuda F Kobayashi T Furihata M Chung S Ashida S Miki T Fujioka T Shuin T Novel lipogenic enzyme ELOVL7 is involved in prostate cancer growth through saturated long-chain fatty acid metabolism Cancer Res 2009 14 8133 8140 10.1158/0008-5472.CAN-09-0775 19826053

[B36] Camps J Pitt JJ Emons G Hummon AB Case CM Grade M Jones TL Nguyen QT Ghadimi BM Beissbarth T Genetic amplification of the NOTCH modulator LNX2 upregulates the WNT/beta-catenin pathway in colorectal cancer Cancer Res 2013 14 2003 2013 10.1158/0008-5472.CAN-12-3159 23319804 PMC4729305

[B37] van Delft FW Horsley S Colman S Anderson K Bateman C Kempski H Zuna J Eckert C Saha V Kearney L Clonal origins of relapse in ETV6-RUNX1 acute lymphoblastic leukemia Blood 2011 14 6247 6254 10.1182/blood-2010-10-314674 21482711

[B38] Miyata Y Liu Y Jankovic V Sashida G Lee JM Shieh JH Naoe T Moore M Nimer SD Cyclin C regulates human hematopoietic stem/progenitor cell quiescence Stem Cells 2010 14 308 317 19967789 10.1002/stem.270 PMC3144254

[B39] Steen H Lindholm D Nuclear localized protein-1 (Nulp1) increases cell death of human osteosarcoma cells and binds the X-linked inhibitor of apoptosis protein Biochem Biophys Res Commun 2008 14 432 437 10.1016/j.bbrc.2007.11.146 18068114

[B40] Veitia RA Bottani S Birchler JA Cellular reactions to gene dosage imbalance: genomic, transcriptomic and proteomic effects Trends Genet 2008 14 390 397 10.1016/j.tig.2008.05.005 18585818

[B41] Vazquez-Mena O Medina-Martinez I Juarez-Torres E Barron V Espinosa A Villegas-Sepulveda N Gomez-Laguna L Nieto-Martinez K Orozco L Roman-Basaure E Amplified genes may be overexpressed, unchanged, or downregulated in cervical cancer cell lines PLoS One 2012 14 e32667 10.1371/journal.pone.0032667 22412903 PMC3296745

[B42] Wang RT Ahn S Park CC Khan AH Lange K Smith DJ Effects of genome-wide copy number variation on expression in mammalian cells BMC Genomics 2011 14 562 10.1186/1471-2164-12-562 22085887 PMC3287593

[B43] Cotter TG Apoptosis and cancer: the genesis of a research field Nat Rev Cancer 2009 14 501 507 10.1038/nrc2663 19550425

