# Supplementary material for: The shaping and functional consequences of the dosage effect landscape in multiple myeloma
Source: BMC Genomics. 2013 Oct 2;14:672. doi: 10.1186/1471-2164-14-672 (PMC3907079; doi:10.1186/1471-2164-14-672)

**Supplementary Tables**

**Suppl. Table 1. Enriched gene ontology groups in the dosage sensitive genes (DES > 0.4) from the IFM dataset.** Only the GO terms with the Benjamini FDR value < 0.05 are shown in this and all following GO analysis tables. Representative GO terms in a cluster are bolded. Count and % in the header indicate the number and percent of genes from the input gene list that belong to a GO term.

| **Enrichment Score** | **GO ID** | **GO Term** | **Count** | **%** | **P-value** | **Benjamini** |
| --- | --- | --- | --- | --- | --- | --- |
| **Cluster 1: 11.12** | 0006414 | translational elongation | 32 | 5.28 | 1.71E-21 | 3.37E-18 |
|  | 0006412 | **translation** | 52 | 8.58 | 9.00E-20 | 8.86E-17 |
|  | 0022626 | cytosolic ribosome | 26 | 4.29 | 4.10E-18 | 1.73E-15 |
|  | 0005840 | ribosome | 36 | 5.94 | 3.49E-15 | 7.26E-13 |
|  | 0033279 | ribosomal subunit | 27 | 4.46 | 6.42E-14 | 6.77E-12 |
|  | 0003735 | structural constituent of ribosome | 30 | 4.95 | 6.82E-13 | 4.37E-10 |
|  | 0044445 | cytosolic part | 27 | 4.46 | 4.26E-12 | 3.59E-10 |
|  | 0022625 | cytosolic large ribosomal subunit | 14 | 2.31 | 1.18E-10 | 6.21E-09 |
|  | 0030529 | ribonucleoprotein complex | 47 | 7.76 | 6.93E-10 | 2.66E-08 |
|  | 0015934 | large ribosomal subunit | 14 | 2.31 | 2.31E-07 | 6.95E-06 |
|  | 0003723 | **RNA binding** | 53 | 8.75 | 3.54E-07 | 1.13E-04 |
|  | 0022627 | cytosolic small ribosomal subunit | 11 | 1.82 | 4.83E-07 | 1.36E-05 |
|  | 0015935 | small ribosomal subunit | 13 | 2.15 | 8.32E-07 | 2.19E-05 |
| **Cluster 2: 9.10** | 0070013 | intracellular organelle lumen | 110 | 18.15 | 3.30E-11 | 2.32E-09 |
|  | 0031974 | membrane-enclosed lumen | 112 | 18.48 | 8.93E-11 | 5.38E-09 |
|  | 0043233 | **organelle lumen** | 110 | 18.15 | 1.31E-10 | 6.16E-09 |
|  | 0005654 | nucleoplasm | 67 | 11.06 | 2.27E-10 | 9.59E-09 |
|  | 0031981 | nuclear lumen | 88 | 14.52 | 1.57E-08 | 5.51E-07 |
|  | 0044451 | nucleoplasm part | 44 | 7.26 | 1.63E-07 | 5.30E-06 |
| **Cluster 3: 4.07** | 0051603 | **proteolysis involved in cellular protein catabolic process** | 44 | 7.26 | 4.60E-06 | 0.001809268 |
|  | 0030163 | protein catabolic process | 45 | 7.43 | 4.80E-06 | 0.001573113 |
|  | 0044257 | cellular protein catabolic process | 44 | 7.26 | 5.14E-06 | 0.001445103 |
|  | 0043632 | modification-dependent macromolecule catabolic process | 42 | 6.93 | 8.19E-06 | 0.001788775 |
|  | 0019941 | modification-dependent protein catabolic process | 42 | 6.93 | 8.19E-06 | 0.001788775 |
|  | 0044265 | cellular macromolecule catabolic process | 48 | 7.92 | 2.31E-05 | 0.004131056 |
|  | 0009057 | macromolecule catabolic process | 49 | 8.09 | 7.10E-05 | 0.011569179 |
|  | 0016879 | ligase activity, forming carbon-nitrogen bonds | 20 | 3.30 | 4.52E-04 | 0.05620032 |
| **Cluster 4: 3.43** | 0005852 | eukaryotic translation initiation factor 3 complex | 7 | 1.16 | 4.86E-06 | 1.14E-04 |
|  | 0006413 | **translational initiation** | 10 | 1.65 | 1.89E-05 | 0.003708109 |
|  | 0008135 | translation factor activity, nucleic acid binding | 13 | 2.15 | 1.44E-04 | 0.030332307 |
| **Cluster 5: 3.24** | 0006461 | **protein complex assembly** | 35 | 5.78 | 1.57E-04 | 0.019079504 |
|  | 0070271 | protein complex biogenesis | 35 | 5.78 | 1.57E-04 | 0.019079504 |
| **Cluster 6: 2.59** | 0008104 | **protein localization** | 58 | 9.57 | 3.12E-06 | 0.002047345 |
|  | 0015031 | protein transport | 52 | 8.58 | 4.04E-06 | 0.001984997 |
|  | 0045184 | establishment of protein localization | 52 | 8.58 | 5.29E-06 | 0.001300585 |
|  | 0034613 | cellular protein localization | 31 | 5.12 | 9.41E-05 | 0.014149769 |
|  | 0006886 | intracellular protein transport | 29 | 4.79 | 1.03E-04 | 0.014430457 |
|  | 0070727 | cellular macromolecule localization | 31 | 5.12 | 1.06E-04 | 0.013827729 |

**Suppl. Table 2. Enriched gene ontology groups in the dosage sensitive genes from the Dickens dataset.** See Suppl. Table 1 legend for description.

| **Enrichment Score** | **GO ID** | **GO Term** | **Count** | **%** | **PValue** | **Benjamini** |
| --- | --- | --- | --- | --- | --- | --- |
| **Cluster 1: 8.16** | 0005739 | **mitochondrion** | 99 | 15.69 | 8.94E-18 | 3.66E-15 |
|  | 0044429 | mitochondrial part | 62 | 9.83 | 1.69E-13 | 3.46E-11 |
|  | 0031090 | organelle membrane | 82 | 13.00 | 4.79E-10 | 6.53E-08 |
|  | 0019866 | organelle inner membrane | 37 | 5.86 | 3.72E-09 | 3.81E-07 |
|  | 0005743 | mitochondrial inner membrane | 34 | 5.39 | 2.44E-08 | 2.00E-06 |
|  | 0070469 | respiratory chain | 16 | 2.54 | 6.70E-08 | 3.91E-06 |
|  | 0031967 | organelle envelope | 51 | 8.08 | 1.07E-07 | 5.46E-06 |
|  | 0031975 | envelope | 51 | 8.08 | 1.18E-07 | 5.39E-06 |
|  | 0006091 | generation of precursor metabolites and energy | 32 | 5.07 | 1.26E-07 | 4.87E-05 |
|  | 0044455 | mitochondrial membrane part | 20 | 3.17 | 1.28E-07 | 5.24E-06 |
|  | 0005740 | mitochondrial envelope | 39 | 6.18 | 2.15E-07 | 7.98E-06 |
|  | 0005746 | mitochondrial respiratory chain | 14 | 2.22 | 4.19E-07 | 1.32E-05 |
|  | 0031966 | mitochondrial membrane | 36 | 5.71 | 1.07E-06 | 2.59E-05 |
|  | 0006119 | oxidative phosphorylation | 15 | 2.38 | 6.29E-06 | 0.001105412 |
| **Cluster 2: 5.80** | 0045333 | **cellular respiration** | 20 | 3.17 | 6.50E-10 | 6.29E-07 |
|  | 0022900 | electron transport chain | 20 | 3.17 | 1.10E-08 | 7.07E-06 |
|  | 0070469 | respiratory chain | 16 | 2.54 | 6.70E-08 | 3.91E-06 |
|  | 0015980 | energy derivation by oxidation of organic compounds | 21 | 3.33 | 1.07E-07 | 5.19E-05 |
|  | 0006091 | generation of precursor metabolites and energy | 32 | 5.07 | 1.26E-07 | 4.87E-05 |
|  | 0044455 | mitochondrial membrane part | 20 | 3.17 | 1.28E-07 | 5.24E-06 |
|  | 0005746 | mitochondrial respiratory chain | 14 | 2.22 | 4.19E-07 | 1.32E-05 |
|  | 0016655 | oxidoreductase activity, acting on NADH or NADPH, quinone or similar compound as acceptor | 12 | 1.90 | 5.57E-07 | 3.67E-04 |
|  | 0006120 | mitochondrial electron transport, NADH to ubiquinone | 11 | 1.74 | 1.17E-06 | 3.76E-04 |
|  | 0050136 | NADH dehydrogenase (quinone) activity | 11 | 1.74 | 1.29E-06 | 4.27E-04 |
|  | 0008137 | NADH dehydrogenase (ubiquinone) activity | 11 | 1.74 | 1.29E-06 | 4.27E-04 |
|  | 0003954 | NADH dehydrogenase activity | 11 | 1.74 | 1.29E-06 | 4.27E-04 |
|  | 0030964 | NADH dehydrogenase complex | 11 | 1.74 | 1.98E-06 | 4.26E-05 |
|  | 0045271 | respiratory chain complex I | 11 | 1.74 | 1.98E-06 | 4.26E-05 |
|  | 0005747 | mitochondrial respiratory chain complex I | 11 | 1.74 | 1.98E-06 | 4.26E-05 |
|  | 0055114 | **oxidation reduction** | 46 | 7.29 | 4.01E-06 | 8.61E-04 |
|  | 0006119 | oxidative phosphorylation | 15 | 2.38 | 6.29E-06 | 0.001105412 |
|  | 0042773 | ATP synthesis coupled electron transport | 11 | 1.74 | 1.83E-05 | 0.002361542 |
|  | 0042775 | mitochondrial ATP synthesis coupled electron transport | 11 | 1.74 | 1.83E-05 | 0.002361542 |
|  | 0016651 | oxidoreductase activity, acting on NADH or NADPH | 13 | 2.06 | 2.21E-05 | 0.003637231 |
|  | 0022904 | respiratory electron transport chain | 11 | 1.74 | 6.09E-05 | 0.006913337 |
| **Cluster 3: 4.45** | 0070013 | intracellular organelle lumen | 105 | 16.64 | 4.29E-07 | 1.25E-05 |
|  | 0031974 | membrane-enclosed lumen | 108 | 17.12 | 5.38E-07 | 1.47E-05 |
|  | 0043233 | **organelle lumen** | 105 | 16.64 | 1.26E-06 | 2.86E-05 |
|  | 0031981 | nuclear lumen | 74 | 11.73 | 0.002690562 | 0.039990371 |
| **Cluster 4: 4.20** | 0070727 | cellular macromolecule localization | 35 | 5.55 | 2.49E-06 | 6.02E-04 |
|  | 0034613 | cellular protein localization | 34 | 5.39 | 5.69E-06 | 0.00110057 |
|  | 0048193 | Golgi vesicle transport | 17 | 2.69 | 1.10E-05 | 0.001635818 |
|  | 0008104 | **protein localization** | 55 | 8.72 | 2.54E-05 | 0.003069256 |
|  | 0046907 | intracellular transport | 43 | 6.81 | 8.27E-05 | 0.008383265 |
|  | 0006886 | intracellular protein transport | 29 | 4.60 | 9.98E-05 | 0.009158541 |
|  | 0045184 | establishment of protein localization | 47 | 7.45 | 1.77E-04 | 0.013106396 |
|  | 0015031 | protein transport | 46 | 7.29 | 2.78E-04 | 0.018388528 |
| **Cluster 5: 4.04** | 0008033 | **tRNA processing** | 13 | 2.06 | 1.01E-05 | 0.001633868 |
|  | 0006399 | tRNA metabolic process | 16 | 2.54 | 1.27E-05 | 0.001753291 |
|  | 0034660 | ncRNA metabolic process | 21 | 3.33 | 1.39E-04 | 0.011106574 |
|  | 0034470 | ncRNA processing | 18 | 2.85 | 2.56E-04 | 0.017558716 |
| **Cluster 6: 3.25** | 0006412 | **translation** | 38 | 6.02 | 2.53E-10 | 4.89E-07 |
|  | 0030529 | ribonucleoprotein complex | 46 | 7.29 | 4.80E-08 | 3.27E-06 |
|  | 0005840 | ribosome | 26 | 4.12 | 3.02E-07 | 1.03E-05 |
|  | 0005759 | mitochondrial matrix | 26 | 4.12 | 8.47E-07 | 2.17E-05 |
|  | 0031980 | mitochondrial lumen | 26 | 4.12 | 8.47E-07 | 2.17E-05 |
|  | 0003735 | structural constituent of ribosome | 19 | 3.01 | 1.62E-05 | 0.003564652 |
|  | 0005761 | mitochondrial ribosome | 9 | 1.43 | 3.12E-04 | 0.006050308 |
|  | 0000313 | organellar ribosome | 9 | 1.43 | 3.12E-04 | 0.006050308 |
|  | 0033279 | ribosomal subunit | 14 | 2.22 | 8.07E-04 | 0.014898916 |
|  | 0015934 | large ribosomal subunit | 9 | 1.43 | 0.002942511 | 0.042131748 |
|  | 0005762 | mitochondrial large ribosomal subunit | 5 | 0.79 | 0.003579174 | 0.049311904 |
|  | 0000315 | organellar large ribosomal subunit | 5 | 0.79 | 0.003579174 | 0.049311904 |

**Suppl. Table 3. Enriched gene ontology groups in differentially expressed genes between HMM and NHMM samples of the IFM dataset.** See Suppl. Table 1 legend for description.

**(A) Up-regulated genes in HMM samples.**

| **Enrichment Score** | **GO ID** | **GO Term** | **Count** | **%** | **PValue** | **Benjamini** |
| --- | --- | --- | --- | --- | --- | --- |
| **Cluster 1: 16.05** | GO:0006414 | translational elongation | 51 | 4.40 | 4.63E-33 | 1.38E-29 |
|  | GO:0022626 | cytosolic ribosome | 39 | 3.36 | 9.85E-25 | 4.64E-22 |
|  | GO:0006412 | **translation** | 78 | 6.73 | 4.50E-24 | 6.70E-21 |
|  | GO:0005840 | ribosome | 59 | 5.09 | 2.57E-22 | 6.04E-20 |
|  | GO:0003735 | structural constituent of ribosome | 52 | 4.49 | 5.67E-22 | 5.63E-19 |
|  | GO:0033279 | ribosomal subunit | 44 | 3.80 | 7.01E-21 | 1.10E-18 |
|  | GO:0044445 | cytosolic part | 42 | 3.62 | 4.96E-16 | 5.23E-14 |
|  | GO:0022625 | cytosolic large ribosomal subunit | 20 | 1.73 | 1.23E-13 | 1.16E-11 |
|  | GO:0030529 | ribonucleoprotein complex | 76 | 6.56 | 3.49E-12 | 2.74E-10 |
|  | GO:0015935 | small ribosomal subunit | 23 | 1.98 | 1.11E-11 | 7.49E-10 |
|  | GO:0022627 | cytosolic small ribosomal subunit | 18 | 1.55 | 6.53E-11 | 3.42E-09 |
|  | GO:0015934 | large ribosomal subunit | 22 | 1.90 | 3.34E-10 | 1.57E-08 |
|  | GO:0005198 | structural molecule activity | 79 | 6.82 | 9.52E-09 | 4.73E-06 |
|  | GO:0003723 | **RNA binding** | 71 | 6.13 | 1.93E-04 | 0.061820094 |

**B) Up-regulated genes in NHMM samples.**

| **Enrichment Score** | **GO ID** | **GO Term** | **Count** | **%** | **PValue** | **Benjamini** |
| --- | --- | --- | --- | --- | --- | --- |
| **Cluster 1: 17.44** | GO:0031974 | membrane-enclosed lumen | 306 | 17.09 | 1.10E-22 | 6.58E-20 |
|  | GO:0031981 | nuclear lumen | 255 | 14.24 | 1.57E-22 | 4.68E-20 |
|  | GO:0070013 | intracellular organelle lumen | 296 | 16.53 | 1.68E-22 | 3.35E-20 |
|  | GO:0043233 | **organelle lumen** | 299 | 16.69 | 7.24E-22 | 1.08E-19 |
|  | GO:0005654 | nucleoplasm | 170 | 9.49 | 8.41E-19 | 1.01E-16 |
|  | GO:0044451 | nucleoplasm part | 107 | 5.97 | 8.18E-12 | 6.11E-10 |
|  | GO:0005730 | nucleolus | 110 | 6.14 | 4.95E-07 | 1.85E-05 |
| **Cluster 2: 12.65** | GO:0008104 | **protein localization** | 167 | 9.32 | 6.11E-17 | 3.84E-13 |
|  | GO:0015031 | protein transport | 150 | 8.38 | 2.04E-16 | 3.84E-13 |
|  | GO:0045184 | establishment of protein localization | 150 | 8.38 | 3.23E-16 | 3.84E-13 |
|  | GO:0046907 | intracellular transport | 128 | 7.15 | 8.28E-14 | 7.17E-11 |
|  | GO:0070727 | cellular macromolecule localization | 85 | 4.75 | 1.37E-10 | 6.80E-08 |
|  | GO:0034613 | cellular protein localization | 84 | 4.69 | 2.27E-10 | 9.81E-08 |
|  | GO:0006886 | intracellular protein transport | 76 | 4.24 | 2.38E-09 | 7.48E-07 |
| **Cluster 3: 8.94** | GO:0015630 | microtubule cytoskeleton | 111 | 6.20 | 1.23E-13 | 1.23E-11 |
|  | GO:0043228 | non-membrane-bounded organelle | 354 | 19.77 | 1.71E-12 | 1.46E-10 |
|  | GO:0043232 | intracellular non-membrane-bounded organelle | 354 | 19.77 | 1.71E-12 | 1.46E-10 |
|  | GO:0005815 | microtubule organizing center | 57 | 3.18 | 3.86E-09 | 2.31E-07 |
|  | GO:0005813 | centrosome | 49 | 2.74 | 1.43E-07 | 6.59E-06 |
|  | GO:0044430 | cytoskeletal part | 139 | 7.76 | 1.15E-06 | 3.82E-05 |
|  | GO:0005856 | **cytoskeleton** | 183 | 10.22 | 1.13E-05 | 3.08E-04 |
| **Cluster 4: 6.97** | GO:0000166 | **nucleotide binding** | 299 | 16.69 | 2.53E-09 | 3.04E-06 |
|  | GO:0032555 | purine ribonucleotide binding | 246 | 13.74 | 6.89E-08 | 2.76E-05 |
|  | GO:0032553 | ribonucleotide binding | 246 | 13.74 | 6.89E-08 | 2.76E-05 |
|  | GO:0017076 | purine nucleotide binding | 255 | 14.24 | 7.03E-08 | 2.11E-05 |
|  | GO:0005524 | ATP binding | 204 | 11.39 | 1.35E-07 | 3.25E-05 |
|  | GO:0001883 | purine nucleoside binding | 216 | 12.06 | 3.23E-07 | 6.47E-05 |
|  | GO:0001882 | nucleoside binding | 217 | 12.12 | 3.58E-07 | 6.13E-05 |
|  | GO:0032559 | adenyl ribonucleotide binding | 204 | 11.39 | 3.68E-07 | 5.52E-05 |
|  | GO:0030554 | adenyl nucleotide binding | 213 | 11.89 | 3.71E-07 | 4.95E-05 |
| **Cluster 5: 6.86** | GO:0030163 | protein catabolic process | 110 | 6.14 | 2.31E-09 | 8.01E-07 |
|  | GO:0044257 | cellular protein catabolic process | 106 | 5.92 | 6.61E-09 | 1.91E-06 |
|  | GO:0009057 | macromolecule catabolic process | 129 | 7.20 | 6.64E-09 | 1.77E-06 |
|  | GO:0051603 | **proteolysis involved in cellular protein catabolic process** | 105 | 5.86 | 1.00E-08 | 2.48E-06 |
|  | GO:0044265 | cellular macromolecule catabolic process | 120 | 6.70 | 2.13E-08 | 4.93E-06 |
|  | GO:0043632 | modification-dependent macromolecule catabolic process | 100 | 5.58 | 2.98E-08 | 6.45E-06 |
|  | GO:0019941 | modification-dependent protein catabolic process | 100 | 5.58 | 2.98E-08 | 6.45E-06 |
|  | GO:0006511 | ubiquitin-dependent protein catabolic process | 42 | 2.35 | 5.01E-04 | 0.035484108 |
| **Cluster 6: 6.26** | GO:0007049 | **cell cycle** | 142 | 7.93 | 4.99E-13 | 3.45E-10 |
|  | GO:0051301 | cell division | 68 | 3.80 | 7.18E-11 | 4.14E-08 |
|  | GO:0000278 | mitotic cell cycle | 71 | 3.96 | 9.40E-08 | 1.71E-05 |
|  | GO:0022402 | cell cycle process | 97 | 5.42 | 1.02E-07 | 1.77E-05 |
|  | GO:0048285 | organelle fission | 45 | 2.51 | 1.51E-05 | 0.002082811 |
|  | GO:0022403 | cell cycle phase | 69 | 3.85 | 2.41E-05 | 0.003199719 |
|  | GO:0007067 | mitosis | 43 | 2.40 | 2.74E-05 | 0.003378763 |
|  | GO:0000280 | nuclear division | 43 | 2.40 | 2.74E-05 | 0.003378763 |
|  | GO:0000087 | M phase of mitotic cell cycle | 43 | 2.40 | 4.25E-05 | 0.004737838 |
|  | GO:0000279 | M phase | 53 | 2.96 | 5.46E-04 | 0.037837109 |
| **Cluster 7: 4.90** | GO:0008134 | **transcription factor binding** | 93 | 5.19 | 5.36E-09 | 3.22E-06 |
|  | GO:0003712 | transcription cofactor activity | 63 | 3.52 | 8.97E-06 | 7.69E-04 |
|  | GO:0016563 | transcription activator activity | 62 | 3.46 | 5.82E-04 | 0.025528313 |
|  | GO:0003713 | transcription coactivator activity | 37 | 2.07 | 8.73E-04 | 0.033253334 |
| **Cluster 8: 4.43** | GO:0006351 | **transcription, DNA-dependent** | 53 | 2.96 | 2.44E-05 | 0.003121041 |
|  | GO:0032774 | RNA biosynthetic process | 53 | 2.96 | 3.56E-05 | 0.004104013 |
|  | GO:0006366 | transcription from RNA polymerase II promoter | 44 | 2.46 | 5.67E-05 | 0.005440892 |
| **Cluster 9: 4.43** | GO:0042623 | ATPase activity, coupled | 54 | 3.02 | 7.56E-07 | 9.07E-05 |
|  | GO:0016887 | **ATPase activity** | 61 | 3.41 | 2.43E-06 | 2.43E-04 |
|  | GO:0004386 | helicase activity | 29 | 1.62 | 1.88E-04 | 0.011818293 |
|  | GO:0008026 | ATP-dependent helicase activity | 22 | 1.23 | 4.42E-04 | 0.022786455 |
|  | GO:0070035 | purine NTP-dependent helicase activity | 22 | 1.23 | 4.42E-04 | 0.022786455 |
| **Cluster 10: 4.12** | GO:0016071 | mRNA metabolic process | 72 | 4.02 | 4.22E-08 | 8.12E-06 |
|  | GO:0006397 | mRNA processing | 61 | 3.41 | 1.17E-06 | 1.84E-04 |
|  | GO:0006396 | **RNA processing** | 88 | 4.91 | 6.73E-06 | 9.70E-04 |
|  | GO:0008380 | RNA splicing | 48 | 2.68 | 3.51E-04 | 0.027866785 |
| **Cluster 11: 4.01** | GO:0016568 | chromatin modification | 53 | 2.96 | 3.80E-06 | 5.71E-04 |
|  | GO:0006325 | chromatin organization | 64 | 3.57 | 2.97E-05 | 0.003541924 |
|  | GO:0051276 | **chromosome organization** | 75 | 4.19 | 1.32E-04 | 0.011967084 |
|  | GO:0016569 | covalent chromatin modification | 26 | 1.45 | 6.08E-04 | 0.04040953 |
|  | GO:0016570 | histone modification | 25 | 1.40 | 8.79E-04 | 0.048649752 |
| **Cluster 12: 3.90** | GO:0004468 | lysine N-acetyltransferase activity | 16 | 0.89 | 1.19E-06 | 1.30E-04 |
|  | GO:0004402 | histone acetyltransferase activity | 16 | 0.89 | 1.19E-06 | 1.30E-04 |
|  | GO:0043543 | protein amino acid acylation | 18 | 1.01 | 5.23E-05 | 0.005158905 |
|  | GO:0008080 | N-acetyltransferase activity | 19 | 1.06 | 8.22E-05 | 0.006144427 |
|  | GO:0016410 | N-acyltransferase activity | 21 | 1.17 | 1.13E-04 | 0.007517037 |
|  | GO:0016573 | histone acetylation | 15 | 0.84 | 1.68E-04 | 0.014818912 |
|  | GO:0000123 | histone acetyltransferase complex | 15 | 0.84 | 2.28E-04 | 0.004382968 |
|  | GO:0006473 | protein amino acid acetylation | 15 | 0.84 | 4.22E-04 | 0.031931346 |
|  | GO:0016569 | covalent chromatin modification | 26 | 1.45 | 6.08E-04 | 0.04040953 |
|  | GO:0016407 | acetyltransferase activity | 19 | 1.06 | 8.18E-04 | 0.032214127 |
|  | GO:0016570 | **histone modification** | 25 | 1.40 | 8.79E-04 | 0.048649752 |
| **Cluster 13: 3.74** | GO:0005694 | chromosome | 75 | 4.19 | 1.26E-05 | 3.27E-04 |
|  | GO:0044427 | chromosomal part | 63 | 3.52 | 6.66E-05 | 0.001372464 |
| **Cluster 14: 3.38** | GO:0033554 | cellular response to stress | 87 | 4.86 | 4.37E-05 | 0.004713136 |
|  | GO:0006974 | **response to DNA damage stimulus** | 58 | 3.24 | 7.15E-04 | 0.044779052 |
|  | GO:0006259 | DNA metabolic process | 74 | 4.13 | 7.65E-04 | 0.046172701 |
| **Cluster 15: 3.08** | GO:0031982 | vesicle | 94 | 5.25 | 3.39E-04 | 0.006119106 |
|  | GO:0031410 | cytoplasmic vesicle | 90 | 5.03 | 4.75E-04 | 0.008076451 |
|  | GO:0016023 | cytoplasmic membrane-bounded vesicle | 77 | 4.30 | 0.001324404 | 0.01826185 |
|  | GO:0031988 | **membrane-bounded vesicle** | 78 | 4.36 | 0.00210631 | 0.025926759 |

**Suppl. Table 4. Enriched gene ontology groups in the dosage resistant genes (DES < 0.2) from the IFM dataset.** See Suppl. Table 1 legend for description.

| **Enrichment Score** | **GO ID** | **GO Term** | **Count** | **%** | **PValue** | **Benjamini** |
| --- | --- | --- | --- | --- | --- | --- |
| **Cluster 1: 8.36** | 0042470 | melanosome | 31 | 3.00 | 3.32E-15 | 1.73E-12 |
|  | 0048770 | pigment granule | 31 | 3.00 | 3.32E-15 | 1.73E-12 |
|  | 0016023 | cytoplasmic membrane-bounded vesicle | 64 | 6.20 | 9.16E-07 | 9.53E-05 |
|  | 0031988 | **membrane-bounded vesicle** | 64 | 6.20 | 2.73E-06 | 1.58E-04 |
|  | 0031410 | cytoplasmic vesicle | 68 | 6.58 | 1.05E-05 | 4.96E-04 |
|  | 0031982 | vesicle | 69 | 6.68 | 2.25E-05 | 9.00E-04 |
| **Cluster 2: 5.01** | 0042981 | **regulation of apoptosis** | 89 | 8.62 | 2.91E-07 | 4.46E-04 |
|  | 0043067 | regulation of programmed cell death | 89 | 8.62 | 4.58E-07 | 4.69E-04 |
|  | 0010941 | regulation of cell death | 89 | 8.62 | 5.40E-07 | 4.14E-04 |
|  | 0006916 | anti-apoptosis | 33 | 3.19 | 2.31E-06 | 0.001417345 |
|  | 0043066 | negative regulation of apoptosis | 41 | 3.97 | 2.89E-04 | 0.03634302 |
|  | 0043069 | negative regulation of programmed cell death | 41 | 3.97 | 3.80E-04 | 0.043816405 |
|  | 0060548 | negative regulation of cell death | 41 | 3.97 | 4.07E-04 | 0.042173276 |
| **Cluster 3: 5.00** | 0042981 | regulation of apoptosis | 89 | 8.62 | 2.91E-07 | 4.46E-04 |
|  | 0043067 | **regulation of programmed cell death** | 89 | 8.62 | 4.58E-07 | 4.69E-04 |
|  | 0010941 | regulation of cell death | 89 | 8.62 | 5.40E-07 | 4.14E-04 |
|  | 0006915 | apoptosis | 64 | 6.20 | 6.14E-05 | 0.016971566 |
|  | 0012501 | programmed cell death | 64 | 6.20 | 9.36E-05 | 0.017793816 |
|  | 0008219 | cell death | 72 | 6.97 | 1.31E-04 | 0.022117225 |
|  | 0016265 | death | 72 | 6.97 | 1.62E-04 | 0.025845428 |
| **Cluster 4: 3.90** | 0016192 | **vesicle-mediated transport** | 71 | 6.87 | 1.08E-07 | 3.30E-04 |
|  | 0016044 | membrane organization | 44 | 4.26 | 1.78E-04 | 0.025697813 |
| **Cluster 5: 3.82** | 0031974 | membrane-enclosed lumen | 161 | 15.59 | 2.00E-06 | 1.73E-04 |
|  | 0043233 | **organelle lumen** | 158 | 15.30 | 2.49E-06 | 1.85E-04 |
|  | 0070013 | intracellular organelle lumen | 155 | 15.00 | 2.68E-06 | 1.74E-04 |
|  | 0005654 | nucleoplasm | 76 | 7.36 | 0.00213725 | 0.043526661 |
|  | 0031981 | nuclear lumen | 115 | 11.13 | 0.002593186 | 0.048777909 |
| **Cluster 6: 3.64** | 0042981 | **regulation of apoptosis** | 89 | 8.62 | 2.91E-07 | 4.46E-04 |
|  | 0043067 | regulation of programmed cell death | 89 | 8.62 | 4.58E-07 | 4.69E-04 |
|  | 0010941 | regulation of cell death | 89 | 8.62 | 5.40E-07 | 4.14E-04 |
| **Cluster 7: 3.63** | 0007264 | small GTPase mediated signal transduction | 42 | 4.07 | 4.29E-06 | 0.002191252 |
|  | 0003924 | **GTPase activity** | 31 | 3.00 | 1.58E-05 | 0.007918529 |
| **Cluster 8: 3.18** | 0006984 | ER-nuclear signaling pathway | 11 | 1.06 | 4.41E-05 | 0.013432232 |
|  | 0051789 | response to protein stimulus | 18 | 1.74 | 4.04E-04 | 0.044902072 |
| **Cluster 9: 3.00** | 0005773 | vacuole | 30 | 2.90 | 7.64E-04 | 0.018754184 |
|  | 0000323 | lytic vacuole | 26 | 2.52 | 0.001139137 | 0.025439884 |
|  | 0005764 | **lysosome** | 26 | 2.52 | 0.001139137 | 0.025439884 |
| **Cluster 10: 2.95** | 0044092 | negative regulation of molecular function | 44 | 4.26 | 7.73E-06 | 0.003383208 |
|  | 0000502 | proteasome complex | 15 | 1.45 | 1.39E-05 | 6.04E-04 |
|  | 0032268 | **regulation of cellular protein metabolic process** | 54 | 5.23 | 4.16E-05 | 0.015833772 |
|  | 0006511 | ubiquitin-dependent protein catabolic process | 33 | 3.19 | 6.57E-05 | 0.016651592 |
|  | 0043086 | negative regulation of catalytic activity | 36 | 3.48 | 7.83E-05 | 0.01701461 |
|  | 0032269 | negative regulation of cellular protein metabolic process | 26 | 2.52 | 1.86E-04 | 0.025559994 |
|  | 0051248 | negative regulation of protein metabolic process | 26 | 2.52 | 3.37E-04 | 0.040583162 |
|  | 0051443 | positive regulation of ubiquitin-protein ligase activity | 14 | 1.36 | 4.12E-04 | 0.041300098 |

**Suppl. Table 5. Enriched gene ontology groups in the dosage resistant genes from the Dickens dataset.** See Suppl. Table 1 legend for description.

| **Enrichment Score** | **GO ID** | **GO Term** | **Count** | **%** | **PValue** | **Benjamini** |
| --- | --- | --- | --- | --- | --- | --- |
| **Cluster 1: 9.03** | GO:0031974 | membrane-enclosed lumen | 158 | 18.90 | 5.75E-13 | 2.60E-10 |
|  | GO:0043233 | **organelle lumen** | 154 | 18.42 | 2.10E-12 | 4.74E-10 |
|  | GO:0070013 | intracellular organelle lumen | 151 | 18.06 | 3.06E-12 | 4.62E-10 |
|  | GO:0031981 | nuclear lumen | 115 | 13.76 | 1.27E-07 | 8.17E-06 |
|  | GO:0005654 | nucleoplasm | 64 | 7.66 | 0.001440353 | 0.027929039 |
| **Cluster 2: 5.90** | GO:0042470 | melanosome | 25 | 2.99 | 3.63E-12 | 4.11E-10 |
|  | GO:0048770 | pigment granule | 25 | 2.99 | 3.63E-12 | 4.11E-10 |
|  | GO:0031988 | **membrane-bounded vesicle** | 48 | 5.74 | 2.78E-04 | 0.010415213 |
|  | GO:0016023 | cytoplasmic membrane-bounded vesicle | 46 | 5.50 | 4.76E-04 | 0.01258909 |
|  | GO:0031982 | vesicle | 52 | 6.22 | 0.00103521 | 0.021055065 |
|  | GO:0031410 | cytoplasmic vesicle | 49 | 5.86 | 0.002147613 | 0.039681438 |
| **Cluster 3: 4.84** | GO:0016265 | death | 65 | 7.78 | 8.69E-06 | 0.003807257 |
|  | GO:0012501 | **programmed cell death** | 57 | 6.82 | 1.18E-05 | 0.004435015 |
|  | GO:0008219 | cell death | 64 | 7.66 | 1.38E-05 | 0.004523033 |
|  | GO:0006915 | apoptosis | 55 | 6.58 | 3.08E-05 | 0.006221929 |
| **Cluster 4: 4.48** | GO:0031980 | mitochondrial lumen | 28 | 3.35 | 1.71E-05 | 8.60E-04 |
|  | GO:0005759 | mitochondrial matrix | 28 | 3.35 | 1.71E-05 | 8.60E-04 |
|  | GO:0044429 | mitochondrial part | 51 | 6.10 | 1.21E-04 | 0.004970852 |
| **Cluster 5: 4.31** | GO:0010941 | **regulation of cell death** | 77 | 9.21 | 1.45E-07 | 1.90E-04 |
|  | GO:0043067 | regulation of programmed cell death | 76 | 9.09 | 2.61E-07 | 2.29E-04 |
|  | GO:0042981 | regulation of apoptosis | 75 | 8.97 | 3.60E-07 | 2.37E-04 |
|  | GO:0006917 | induction of apoptosis | 36 | 4.31 | 1.51E-05 | 0.00441051 |
|  | GO:0012502 | induction of programmed cell death | 36 | 4.31 | 1.62E-05 | 0.004253877 |
|  | GO:0043065 | positive regulation of apoptosis | 40 | 4.78 | 3.12E-04 | 0.03511149 |
|  | GO:0060548 | negative regulation of cell death | 35 | 4.19 | 3.56E-04 | 0.035399993 |
|  | GO:0043068 | positive regulation of programmed cell death | 40 | 4.78 | 3.58E-04 | 0.034355654 |
|  | GO:0010942 | positive regulation of cell death | 40 | 4.78 | 3.96E-04 | 0.035365806 |
| **Cluster 6: 3.78** | GO:0000166 | **nucleotide binding** | 162 | 19.38 | 7.99E-07 | 7.52E-04 |
|  | GO:0017076 | purine nucleotide binding | 134 | 16.03 | 5.13E-05 | 0.015956164 |
|  | GO:0032555 | purine ribonucleotide binding | 129 | 15.43 | 5.88E-05 | 0.013747385 |
|  | GO:0032553 | ribonucleotide binding | 129 | 15.43 | 5.88E-05 | 0.013747385 |
| **Cluster 7: 3.37** | GO:0070647 | protein modification by small protein conjugation or removal | 23 | 2.75 | 1.89E-05 | 0.004141035 |
|  | GO:0044265 | cellular macromolecule catabolic process | 63 | 7.54 | 3.47E-05 | 0.006510244 |
|  | GO:0030163 | protein catabolic process | 56 | 6.70 | 3.99E-05 | 0.006979214 |
|  | GO:0051603 | **proteolysis involved in cellular protein catabolic process** | 54 | 6.46 | 5.47E-05 | 0.00896006 |
|  | GO:0044257 | cellular protein catabolic process | 54 | 6.46 | 6.38E-05 | 0.009839603 |
|  | GO:0009057 | macromolecule catabolic process | 65 | 7.78 | 8.81E-05 | 0.0128218 |
|  | GO:0019941 | modification-dependent protein catabolic process | 51 | 6.10 | 1.27E-04 | 0.017483423 |
|  | GO:0043632 | modification-dependent macromolecule catabolic process | 51 | 6.10 | 1.27E-04 | 0.017483423 |
| **Cluster 8: 2.98** | GO:0016604 | nuclear body | 22 | 2.63 | 7.08E-05 | 0.003195508 |
|  | GO:0016607 | nuclear speck | 15 | 1.79 | 4.73E-04 | 0.013273062 |
| **Cluster 9: 2.73** | GO:0006412 | **translation** | 47 | 5.62 | 4.14E-10 | 1.09E-06 |
|  | GO:0030529 | ribonucleoprotein complex | 53 | 6.34 | 4.86E-07 | 2.74E-05 |
|  | GO:0005840 | ribosome | 23 | 2.75 | 8.50E-04 | 0.018134664 |
| **Cluster 10: 2.61** | GO:0010008 | endosome membrane | 11 | 1.32 | 3.91E-04 | 0.012545025 |
|  | GO:0044440 | endosomal part | 11 | 1.32 | 3.91E-04 | 0.012545025 |

**Suppl. Table 6. Enriched gene ontology groups in the dosage sensitive genes (DES > 0.4) from the Aroma.Affymetrix preprocessed IFM dataset.** See Suppl. Table 1 legend for description.

| **Category** | **GO ID** | **GO Term** | **Count** | **%** | **PValue** | **Benjamini** |
| --- | --- | --- | --- | --- | --- | --- |
| **Cluster 1: 24.66** | GO:0070013 | intracellular organelle lumen | 292 | 19.17 | 2.79E-33 | 1.59E-30 |
|  | GO:0043233 | organelle lumen | 292 | 19.17 | 1.87E-31 | 5.34E-29 |
|  | GO:0031974 | membrane-enclosed lumen | 295 | 19.37 | 4.44E-31 | 8.45E-29 |
|  | GO:0031981 | nuclear lumen | 233 | 15.30 | 1.46E-24 | 2.09E-22 |
|  | GO:0005654 | nucleoplasm | 150 | 9.85 | 8.42E-18 | 6.01E-16 |
|  | GO:0044451 | nucleoplasm part | 102 | 6.70 | 3.38E-14 | 1.93E-12 |
| **Cluster 2: 12.81** | GO:0006412 | translation | 102 | 6.70 | 2.84E-31 | 8.37E-28 |
|  | GO:0030529 | ribonucleoprotein complex | 113 | 7.42 | 6.03E-22 | 6.89E-20 |
|  | GO:0005840 | ribosome | 65 | 4.27 | 3.34E-20 | 2.72E-18 |
|  | GO:0006414 | translational elongation | 44 | 2.89 | 3.36E-20 | 4.95E-17 |
|  | GO:0022626 | cytosolic ribosome | 33 | 2.17 | 1.50E-14 | 9.51E-13 |
|  | GO:0003735 | structural constituent of ribosome | 49 | 3.22 | 2.87E-14 | 2.98E-11 |
|  | GO:0033279 | ribosomal subunit | 40 | 2.63 | 4.13E-13 | 1.96E-11 |
|  | GO:0044445 | cytosolic part | 39 | 2.56 | 5.58E-10 | 1.99E-08 |
|  | GO:0015935 | small ribosomal subunit | 21 | 1.38 | 1.02E-07 | 3.24E-06 |
|  | GO:0022625 | cytosolic large ribosomal subunit | 16 | 1.05 | 1.58E-07 | 4.76E-06 |
|  | GO:0015934 | large ribosomal subunit | 20 | 1.31 | 1.49E-06 | 4.25E-05 |
|  | GO:0022627 | cytosolic small ribosomal subunit | 15 | 0.98 | 2.28E-06 | 5.41E-05 |
| **Cluster 3: 8.72** | GO:0043228 | non-membrane-bounded organelle | 315 | 20.68 | 1.55E-13 | 8.03E-12 |
|  | GO:0043232 | intracellular non-membrane-bounded organelle | 315 | 20.68 | 1.55E-13 | 8.03E-12 |
| **Cluster 4: 8.17** | GO:0044429 | mitochondrial part | 97 | 6.37 | 1.97E-10 | 7.51E-09 |
|  | GO:0005759 | mitochondrial matrix | 46 | 3.02 | 3.90E-08 | 1.31E-06 |
|  | GO:0031980 | mitochondrial lumen | 46 | 3.02 | 3.90E-08 | 1.31E-06 |
| **Cluster 5: 7.92** | GO:0008104 | protein localization | 137 | 9.00 | 5.50E-12 | 5.40E-09 |
|  | GO:0015031 | protein transport | 122 | 8.01 | 1.33E-11 | 9.83E-09 |
|  | GO:0045184 | establishment of protein localization | 122 | 8.01 | 2.45E-11 | 1.44E-08 |
|  | GO:0046907 | intracellular transport | 103 | 6.76 | 2.16E-09 | 7.96E-07 |
|  | GO:0070727 | cellular macromolecule localization | 72 | 4.73 | 1.17E-08 | 3.84E-06 |
|  | GO:0034613 | cellular protein localization | 70 | 4.60 | 4.60E-08 | 1.23E-05 |
|  | GO:0006886 | intracellular protein transport | 64 | 4.20 | 1.56E-07 | 3.53E-05 |
|  | GO:0006605 | protein targeting | 43 | 2.82 | 3.54E-07 | 6.95E-05 |
|  | GO:0017038 | protein import | 30 | 1.97 | 1.69E-06 | 2.16E-04 |
|  | GO:0033365 | protein localization in organelle | 27 | 1.77 | 2.81E-04 | 0.018643723 |
| **Cluster 6: 5.91** | GO:0005739 | mitochondrion | 159 | 10.44 | 1.14E-12 | 5.00E-11 |
|  | GO:0044429 | mitochondrial part | 97 | 6.37 | 1.97E-10 | 7.51E-09 |
|  | GO:0031967 | organelle envelope | 87 | 5.71 | 1.84E-06 | 5.01E-05 |
|  | GO:0031090 | organelle membrane | 136 | 8.93 | 2.05E-06 | 5.33E-05 |
|  | GO:0031975 | envelope | 87 | 5.71 | 2.11E-06 | 5.24E-05 |
|  | GO:0044455 | mitochondrial membrane part | 27 | 1.77 | 1.17E-05 | 2.47E-04 |
|  | GO:0031966 | mitochondrial membrane | 57 | 3.74 | 6.08E-05 | 0.001051054 |
|  | GO:0005740 | mitochondrial envelope | 59 | 3.87 | 9.52E-05 | 0.0015093 |
|  | GO:0005743 | mitochondrial inner membrane | 46 | 3.02 | 1.44E-04 | 0.002213763 |
|  | GO:0019866 | organelle inner membrane | 47 | 3.09 | 3.91E-04 | 0.005713307 |
| **Cluster 7: 5.67** | GO:0034660 | ncRNA metabolic process | 52 | 3.41 | 2.03E-10 | 9.98E-08 |
|  | GO:0006399 | tRNA metabolic process | 31 | 2.04 | 4.40E-08 | 1.30E-05 |
|  | GO:0034470 | ncRNA processing | 40 | 2.63 | 1.57E-07 | 3.30E-05 |
|  | GO:0022613 | ribonucleoprotein complex biogenesis | 38 | 2.50 | 4.74E-07 | 7.76E-05 |
|  | GO:0042254 | ribosome biogenesis | 29 | 1.90 | 1.19E-06 | 1.66E-04 |
|  | GO:0008033 | tRNA processing | 18 | 1.18 | 2.02E-04 | 0.014760538 |
|  | GO:0016072 | rRNA metabolic process | 19 | 1.25 | 0.001263882 | 0.061175644 |
|  | GO:0006364 | rRNA processing | 18 | 1.18 | 0.002008115 | 0.084564958 |
| **Cluster 8: 5.45** | GO:0044265 | cellular macromolecule catabolic process | 106 | 6.96 | 5.39E-08 | 1.32E-05 |
|  | GO:0009057 | macromolecule catabolic process | 109 | 7.16 | 3.80E-07 | 7.00E-05 |
|  | GO:0051603 | proteolysis involved in cellular protein catabolic process | 89 | 5.84 | 3.92E-07 | 6.80E-05 |
|  | GO:0044257 | cellular protein catabolic process | 89 | 5.84 | 4.92E-07 | 7.63E-05 |
|  | GO:0030163 | protein catabolic process | 91 | 5.98 | 5.31E-07 | 7.82E-05 |
|  | GO:0019941 | modification-dependent protein catabolic process | 84 | 5.52 | 1.52E-06 | 2.04E-04 |
|  | GO:0043632 | modification-dependent macromolecule catabolic process | 84 | 5.52 | 1.52E-06 | 2.04E-04 |
| **Cluster 9: 4.95** | GO:0008135 | translation factor activity, nucleic acid binding | 31 | 2.04 | 3.12E-10 | 1.08E-07 |
|  | GO:0005852 | eukaryotic translation initiation factor 3 complex | 9 | 0.59 | 8.99E-06 | 1.97E-04 |
|  | GO:0003743 | translation initiation factor activity | 17 | 1.12 | 3.78E-05 | 0.003009637 |
|  | GO:0006413 | translational initiation | 14 | 0.92 | 7.16E-05 | 0.006180271 |
| **Cluster 10: 4.61** | GO:0008134 | transcription factor binding | 77 | 5.06 | 1.37E-06 | 2.84E-04 |
|  | GO:0003713 | transcription coactivator activity | 40 | 2.63 | 4.74E-06 | 8.18E-04 |
|  | GO:0003712 | transcription cofactor activity | 57 | 3.74 | 1.03E-05 | 0.001182109 |
|  | GO:0016563 | transcription activator activity | 61 | 4.01 | 2.60E-05 | 0.00244351 |
| **Cluster 11: 4.47** | GO:0006399 | tRNA metabolic process | 31 | 2.04 | 4.40E-08 | 1.30E-05 |
|  | GO:0043038 | amino acid activation | 14 | 0.92 | 9.20E-05 | 0.007710114 |
|  | GO:0043039 | tRNA aminoacylation | 14 | 0.92 | 9.20E-05 | 0.007710114 |
|  | GO:0006418 | tRNA aminoacylation for protein translation | 14 | 0.92 | 9.20E-05 | 0.007710114 |
|  | GO:0016875 | ligase activity, forming carbon-oxygen bonds | 14 | 0.92 | 1.11E-04 | 0.007146549 |
|  | GO:0016876 | ligase activity, forming aminoacyl-tRNA and related compounds | 14 | 0.92 | 1.11E-04 | 0.007146549 |
|  | GO:0004812 | aminoacyl-tRNA ligase activity | 14 | 0.92 | 1.11E-04 | 0.007146549 |
| **Cluster 12: 4.39** | GO:0016879 | ligase activity, forming carbon-nitrogen bonds | 45 | 2.95 | 3.34E-07 | 8.65E-05 |
|  | GO:0016881 | acid-amino acid ligase activity | 38 | 2.50 | 6.50E-06 | 9.62E-04 |
|  | GO:0019787 | small conjugating protein ligase activity | 33 | 2.17 | 1.01E-05 | 0.00131307 |
|  | GO:0000151 | ubiquitin ligase complex | 22 | 1.44 | 1.27E-05 | 2.60E-04 |
|  | GO:0004842 | ubiquitin-protein ligase activity | 28 | 1.84 | 1.16E-04 | 0.007033565 |
|  | GO:0016567 | protein ubiquitination | 23 | 1.51 | 4.73E-04 | 0.030472414 |
|  | GO:0070647 | protein modification by small protein conjugation or removal | 28 | 1.84 | 5.27E-04 | 0.033198683 |
|  | GO:0032446 | protein modification by small protein conjugation | 24 | 1.58 | 8.43E-04 | 0.04579076 |
| **Cluster 13: 3.94** | GO:0006605 | protein targeting | 43 | 2.82 | 3.54E-07 | 6.95E-05 |
|  | GO:0017038 | protein import | 30 | 1.97 | 1.69E-06 | 2.16E-04 |
|  | GO:0051169 | nuclear transport | 32 | 2.10 | 1.08E-05 | 0.001266339 |
|  | GO:0006913 | nucleocytoplasmic transport | 31 | 2.04 | 2.23E-05 | 0.002519807 |
|  | GO:0033365 | protein localization in organelle | 27 | 1.77 | 2.81E-04 | 0.018643723 |
| **Cluser 14: 3.86** | GO:0070271 | protein complex biogenesis | 72 | 4.73 | 2.30E-05 | 0.00251041 |
|  | GO:0006461 | protein complex assembly | 72 | 4.73 | 2.30E-05 | 0.00251041 |
|  | GO:0065003 | macromolecular complex assembly | 88 | 5.78 | 4.54E-05 | 0.004594919 |
|  | GO:0043933 | macromolecular complex subunit organization | 92 | 6.04 | 6.68E-05 | 0.005944361 |
| **Cluster 15: 3.61** | GO:0009108 | coenzyme biosynthetic process | 18 | 1.18 | 5.61E-05 | 0.005489796 |
|  | GO:0051188 | cofactor biosynthetic process | 21 | 1.38 | 1.89E-04 | 0.014176127 |
|  | GO:0006732 | coenzyme metabolic process | 28 | 1.84 | 2.52E-04 | 0.01754896 |
| **Cluster 16: 3.56** | GO:0006351 | transcription, DNA-dependent | 47 | 3.09 | 4.21E-05 | 0.004413743 |
|  | GO:0032774 | RNA biosynthetic process | 47 | 3.09 | 5.92E-05 | 0.005609134 |
|  | GO:0006366 | transcription from RNA polymerase II promoter | 38 | 2.50 | 2.15E-04 | 0.015312994 |
|  | GO:0006352 | transcription initiation | 18 | 1.18 | 6.06E-04 | 0.035783233 |
| **Cluster 17: 3.35** | GO:0000166 | nucleotide binding | 241 | 15.82 | 6.54E-05 | 0.004509494 |
|  | GO:0030554 | adenyl nucleotide binding | 175 | 11.49 | 1.76E-04 | 0.010065662 |
|  | GO:0001883 | purine nucleoside binding | 176 | 11.56 | 2.65E-04 | 0.012994241 |
|  | GO:0005524 | ATP binding | 164 | 10.77 | 2.86E-04 | 0.013377245 |
|  | GO:0032559 | adenyl ribonucleotide binding | 165 | 10.83 | 3.82E-04 | 0.017067557 |
|  | GO:0001882 | nucleoside binding | 176 | 11.56 | 3.97E-04 | 0.016984919 |
|  | GO:0017076 | purine nucleotide binding | 202 | 13.26 | 8.62E-04 | 0.03032711 |
| **Cluster 18: 3.35** | GO:0006396 | RNA processing | 93 | 6.11 | 2.16E-10 | 9.08E-08 |
|  | GO:0016071 | mRNA metabolic process | 54 | 3.55 | 1.53E-04 | 0.012085962 |
|  | GO:0008380 | RNA splicing | 42 | 2.76 | 7.06E-04 | 0.039968431 |
|  | GO:0006397 | mRNA processing | 46 | 3.02 | 7.41E-04 | 0.041111196 |
| **Cluster 19: 3.26** | GO:0015630 | microtubule cytoskeleton | 77 | 5.06 | 8.04E-06 | 1.84E-04 |
|  | GO:0005813 | centrosome | 39 | 2.56 | 2.11E-05 | 4.02E-04 |
|  | GO:0005815 | microtubule organizing center | 41 | 2.69 | 6.78E-05 | 0.001106107 |
| **Cluster 20: 3.14** | GO:0051169 | nuclear transport | 32 | 2.10 | 1.08E-05 | 0.001266339 |
|  | GO:0006913 | nucleocytoplasmic transport | 31 | 2.04 | 2.23E-05 | 0.002519807 |
|  | GO:0006403 | RNA localization | 22 | 1.44 | 1.00E-04 | 0.008159654 |
|  | GO:0050658 | RNA transport | 20 | 1.31 | 5.36E-04 | 0.033036796 |
|  | GO:0050657 | nucleic acid transport | 20 | 1.31 | 5.36E-04 | 0.033036796 |
|  | GO:0051236 | establishment of RNA localization | 20 | 1.31 | 5.36E-04 | 0.033036796 |
|  | GO:0005643 | nuclear pore | 17 | 1.12 | 7.40E-04 | 0.010507899 |

**Suppl. Table 7. Enriched gene ontology groups in the dosage resistant genes (DES < 0.2) from the Aroma.Affymetrix preprocessed IFM dataset.** See Suppl. Table 1 legend for description.

| **Category** | **GO ID** | **GO Term** | **Count** | **%** | **PValue** | **Benjamini** |
| --- | --- | --- | --- | --- | --- | --- |
| **Cluster 1: 6.67** | GO:0042470 | melanosome | 19 | 3.42 | 4.22E-10 | 1.85E-07 |
|  | GO:0048770 | pigment granule | 19 | 3.42 | 4.22E-10 | 1.85E-07 |
|  | GO:0031988 | membrane-bounded vesicle | 41 | 7.37 | 3.49E-06 | 2.18E-04 |
|  | GO:0016023 | cytoplasmic membrane-bounded vesicle | 40 | 7.19 | 3.99E-06 | 2.18E-04 |
|  | GO:0031410 | cytoplasmic vesicle | 44 | 7.91 | 5.35E-06 | 2.60E-04 |
|  | GO:0031982 | vesicle | 45 | 8.09 | 6.80E-06 | 2.97E-04 |
| **Cluster 2: 5.23** | GO:0007264 | small GTPase mediated signal transduction | 34 | 6.12 | 4.73E-09 | 1.21E-05 |
|  | GO:0003924 | GTPase activity | 21 | 3.78 | 1.76E-05 | 0.012118027 |
|  | GO:0005525 | GTP binding | 29 | 5.22 | 3.14E-05 | 0.010816823 |
|  | GO:0019001 | guanyl nucleotide binding | 29 | 5.22 | 5.04E-05 | 0.008696515 |
|  | GO:0032561 | guanyl ribonucleotide binding | 29 | 5.22 | 5.04E-05 | 0.008696515 |
| **Cluster 3: 4.64** | GO:0042981 | regulation of apoptosis | 60 | 10.79 | 2.05E-08 | 2.63E-05 |
|  | GO:0043067 | regulation of programmed cell death | 60 | 10.79 | 2.88E-08 | 2.46E-05 |
|  | GO:0010941 | regulation of cell death | 60 | 10.79 | 3.30E-08 | 2.11E-05 |
|  | GO:0008219 | cell death | 50 | 8.99 | 3.02E-06 | 0.001105128 |
|  | GO:0016265 | death | 50 | 8.99 | 3.64E-06 | 0.001163732 |
|  | GO:0043066 | negative regulation of apoptosis | 31 | 5.58 | 4.65E-06 | 0.001322792 |
|  | GO:0043069 | negative regulation of programmed cell death | 31 | 5.58 | 6.12E-06 | 0.001565929 |
|  | GO:0060548 | negative regulation of cell death | 31 | 5.58 | 6.48E-06 | 0.00150782 |
|  | GO:0006916 | anti-apoptosis | 22 | 3.96 | 8.04E-06 | 0.001713954 |
|  | GO:0006915 | apoptosis | 42 | 7.55 | 2.08E-05 | 0.004093447 |
|  | GO:0012501 | programmed cell death | 42 | 7.55 | 2.93E-05 | 0.005342647 |
| **Cluster 4: 4.24** | GO:0010035 | response to inorganic substance | 23 | 4.14 | 2.10E-06 | 0.001073636 |
|  | GO:0010038 | response to metal ion | 16 | 2.88 | 3.45E-05 | 0.005865545 |
| **Cluster 5: 3.89** | GO:0000267 | cell fraction | 65 | 11.69 | 1.82E-06 | 1.33E-04 |
|  | GO:0005624 | membrane fraction | 44 | 7.91 | 0.001001777 | 0.018084306 |
|  | GO:0005626 | insoluble fraction | 45 | 8.09 | 0.001155295 | 0.018535535 |
| **Cluster 6: 3.95** | GO:0005773 | vacuole | 21 | 3.78 | 2.21E-04 | 0.005072511 |
|  | GO:0005764 | lysosome | 18 | 3.24 | 5.44E-04 | 0.010740724 |
|  | GO:0000323 | lytic vacuole | 18 | 3.24 | 5.44E-04 | 0.010740724 |
| **Cluster 7: 3.37** | GO:0031974 | membrane-enclosed lumen | 92 | 16.55 | 2.64E-05 | 9.59E-04 |
|  | GO:0043233 | organelle lumen | 90 | 16.19 | 3.65E-05 | 0.001138315 |
|  | GO:0070013 | intracellular organelle lumen | 88 | 15.83 | 4.77E-05 | 0.001303196 |
|  | GO:0031981 | nuclear lumen | 70 | 12.59 | 7.13E-04 | 0.013451976 |
|  | GO:0005730 | nucleolus | 39 | 7.01 | 0.001284499 | 0.019182151 |
| **Cluster 8: 2.82** | GO:0030176 | integral to endoplasmic reticulum membrane | 9 | 1.62 | 1.21E-04 | 0.003116078 |
|  | GO:0044432 | endoplasmic reticulum part | 26 | 4.68 | 1.76E-04 | 0.004253237 |
|  | GO:0031227 | intrinsic to endoplasmic reticulum membrane | 9 | 1.62 | 0.001055619 | 0.018292591 |
|  | GO:0042175 | nuclear envelope-endoplasmic reticulum network | 20 | 3.60 | 0.002428647 | 0.031687382 |
|  | GO:0031300 | intrinsic to organelle membrane | 13 | 2.34 | 0.002916137 | 0.034829378 |
| **Cluster 9: 2.70** | GO:0010008 | endosome membrane | 9 | 1.62 | 4.80E-04 | 0.009948464 |
|  | GO:0044440 | endosomal part | 9 | 1.62 | 4.80E-04 | 0.009948464 |
|  | GO:0005765 | lysosomal membrane | 6 | 1.08 | 0.001591598 | 0.022935635 |
|  | GO:0005774 | vacuolar membrane | 8 | 1.44 | 0.002153389 | 0.029931473 |
|  | GO:0044437 | vacuolar part | 8 | 1.44 | 0.004620742 | 0.04933981 |
| **Cluster 10: 2.53** | GO:0010033 | response to organic substance | 42 | 7.55 | 9.67E-04 | 0.090877486 |

**Suppl. Figure 1. Overlap of functional enrichment analysis results.**


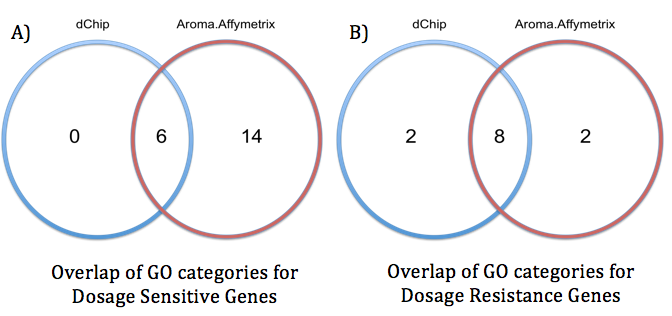


**Suppl. Figure 2. The CNA frequency and dosage effect karyotype plot for the IFM dataset from Aroma.Affymetrix platform.**


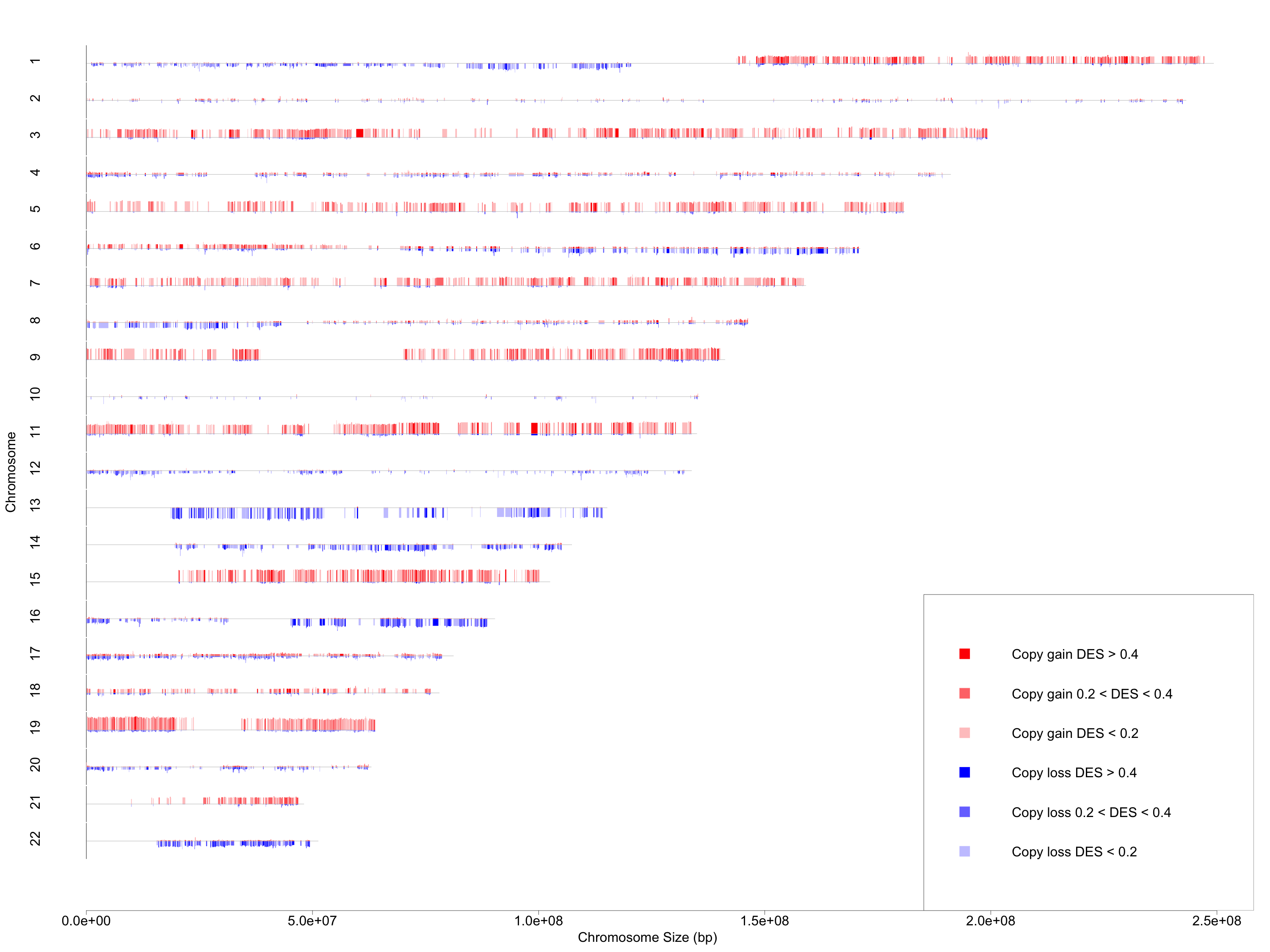

Supplement: Additional file 5 — Supplementary tables and figures. [file 1471-2164-14-672-S5.docx]
